# Regular Physical Activity, Short-Term Exercise, Mental Health, and Well-Being Among University Students: The Results of an Online and a Laboratory Study

**DOI:** 10.3389/fpsyg.2020.00509

**Published:** 2020-05-26

**Authors:** Cornelia Herbert, Friedrich Meixner, Christine Wiebking, Verena Gilg

**Affiliations:** Department of Applied Emotion and Motivation Psychology, Institute of Psychology and Education, Ulm University, Ulm, Germany

**Keywords:** physical activity, aerobic exercise, cardiovascular fitness, mental health, depression, stress, well-being, university students

## Abstract

The health benefits of regular physical activity and aerobic exercise are undisputed in the literature. The present series of pilot studies had two major objectives: (a) examine mental health, well-being, and regular physical activity of university students and (b) explore the potential health benefits of short-term aerobic exercise on university students in an online and a laboratory study. Mental health and well-being were measured before (Time 1, T1) and after (Time 2, T2) a 6 week (online study) and 2 week (laboratory study) low- to moderate-intensity aerobic exercise intervention. Mental health and well-being were assessed using standardized self-report measures of depression, anxiety, positive and negative affect, perceived stress and coping strategies, body dissatisfaction, and quality of life. The effects of the aerobic exercise were compared to a cognitive non-exercise control condition (online study), motor coordination exercise (laboratory study), and a waiting list (online and laboratory). A total of 185 university students were recruited from German universities at T1. Further, 74 (women: *n* = 67) students completed the 6-week intervention. Similarly, 32 (women: *n* = 30) participants completed the 2 week intervention (laboratory study). At T1, 36.6% of the students (women and men) reported experiencing depressive symptoms. 41.83% of them (women and men) had high levels of state anxiety. All the students reported experiencing stress (e.g., due to uncertainty related to factors such as their finances, job, and social relationships). At T1, regular physical activity was negatively correlated with self-reported depression, anxiety, and perceived psychosomatic stress and positively correlated with quality of life and positive affect. Among women, cardiovascular fitness (operationalized as resting heart rate variability) was negatively correlated with self-reported anxiety (state) and depression at T1 (laboratory study). The 6 week aerobic exercise intervention resulted in significant improvements in self-reported depression, overall perceived stress, and perceived stress due to uncertainty. The present results confirm that there is a relationship between regular physical activity, cardiovascular fitness, mental health, and well-being among university students. They support the hypothesis that short-term aerobic exercise interventions can act as buffer against depression and perceived stress in university students after 6 weeks of aerobic exercise of low to moderate intensity.

## Introduction

### Health Benefits of Regular Physical Activity and Exercise

The health benefits of regular physical activity and exercise are undisputed in the literature. Particularly large health benefits have been reported among individuals who have experienced significant losses in their psychological and physical functions as a result of chronic diseases such as cardiovascular disease, diabetes, cancer, hypertension, obesity, depression, and osteoporosis (for an overview, see Taylor et al., 1985; Paluska and Schwenk, 2000; Penedo and Dahn, 2005; Haskell et al., 2007; Knapen et al., 2015; Ruegsegger and Booth, 2018). Among such patient groups whose symptom severity requires secondary and tertiary prevention, significant improvements in cardiovascular fitness and self-reported mood, anxiety, and depressive symptoms can be achieved by regularly engaging in physical activity. Regular physical activity and exercise have also been found to result in lower vulnerability to psychological stressors during periods of increased workload and cognitive performance under pressure among these patient groups (Norris et al., 1992; Stults-Kolehmainen and Sinha, 2014).

Past studies have demonstrated significant effects of aerobic exercise (e.g., endurance exercises such as walking, swimming, treadmill running, and cycling) on mental and physical health indicators using quasi-experimental between-subjects (i.e., comparisons of physically active and sedentary control groups) and within-subjects designs (i.e., comparisons of the same groups before and after exercise interventions). However, the exercise intensity and duration at which the health-related effects of exercise reach statistical significance have been found to vary substantially across studies, samples (i.e., patients vs. healthy participants), and people of different ages and gender (for an overview, see Gerber and Pühse, 2009; Stoll and Ziemainz, 2012). The observed effects may also depend on the methods used to measure exercise-related health gains (e.g., self-report, behavioral, neurophysiological).

According to the international recommendations of the World Health Organization (WHO) and American College of Sports Medicine (ACSM), healthy adults should engage in (a) moderate-intensity aerobic exercise (∼75% of the maximal oxygen consumption (VO_2_ max) = 3–6 metabolic equivalents (METs)) for 30 min a day, 5 days a week, or (b) vigorous-intensity aerobic exercise (>75% of the VO_2_ max > 6 METs) for 20 min a day, 3 days a week to maintain their physical health. Further, additional bouts of exercise have been recommended to reduce the lifetime risk for chronic diseases or unhealthy weight gain and improve cardiovascular fitness (Haskell et al., 2007; World Health Organization [WHO], 2010; American College of Sports Medicine, 2013).

The aforementioned recommendations of the WHO and ACSM are based on global epidemiologic health indicators (e.g., morbidity rates, mortality risk) or lifetime risk for certain somatic diseases including cancer and cardiovascular diseases. The question of whether the same exercise recommendations are valid for the prevention of mental health and well-being continues to be debated.

### Effects of Exercise on Mental Health and Well-Being

In the literature, the terms *mental health* and *well-being* are used as umbrella terms to refer to psychological, mental, cognitive, and affective factors that enhance or impair the functioning of a person. Accordingly, psychological, subjective, and overall well-being have been identified as major contributors to mental health. This perspective concurs with theoretical definitions of well-being, which emphasize the absence of mental and physical illness (for reviews, see Diener and Ryan, 2009). Psychological models and theories of well-being correspondingly emphasize that well-being is related to optimal experiences and functioning of both the body and mind (e.g., Ryan and Deci, 2001). Consequently, past studies (including meta-analytic studies and reviews) on the relationships between physical activity, exercise, mental health, and well-being have investigated the effects of physical activity and exercise on a broad range of variables including depression, anxiety, mood/affect, stress, body image/body dissatisfaction, and quality of life (Scully et al., 1998; Biddle et al., 2000; Paluska and Schwenk, 2000). In the remainder of this manuscript, the broader term well-being will be used to refer to the positive effects that exercise has on the factors (i.e., anxiety, depression, and perceived stress) that negatively impact mental health and well-being.

#### Self-Reported Anxiety

In their meta-analytic study, Petruzzello et al. (1991) summarized the results of more than 124 studies that had investigated the effects of acute and regular aerobic and anaerobic exercise on mental health, well-being, and anxiety, in particular (Petruzzello et al., 1991). The results revealed that only moderate- to high-intensity aerobic exercise had resulted in significant changes in self-reported anxiety symptoms. Further, these effects were independent of gender, age, and physical health status. Recent meta-analytic findings suggest that acute bouts of exercise can reduce state anxiety (Ensari et al., 2015). Aerobic exercise exceeding 21 min is associated with significant anxiolytic effects on self-reported state and trait anxiety after 10 weeks of regular exercise. However, effect sizes for exercise-induced psychophysiological changes that are related to anxiety symptoms have been found to be much smaller than those reported for self-report measures (Petruzzello et al., 1991). Recent meta-analytic findings support the notion that aerobic exercise is particularly effective in reducing anxiety among non-clinical samples, but the effect sizes have been found to be small (Rebar et al., 2015). Concerning clinically relevant anxiety symptoms, exercise interventions do not have the same effects that psychopharmaceutic treatments for anxiety do (Carek et al., 2011). However, aerobic exercise has been found to be effective in alleviating several anxiety (e.g., generalized anxiety, panic, obsessive-compulsive disorder, social phobia) and stress-related disorders (posttraumatic stress disorder; Stubbs et al., 2017).

#### Self-Reported Depression

The largest changes in self-reported depressive symptoms appear to result from engagement in moderate- to vigorous-intensity aerobic exercise. The acute antidepressive effects of aerobic exercise have been confirmed in clinical randomized controlled trials in which depressive patients were assigned to receive either an exercise intervention, psychotherapy, or both (for recent meta-analyses e.g., Schuch et al., 2016; Morres et al., 2019). Thus, the effects of regular aerobic exercise on self-reported depressive symptoms can be as strong as those of psychotherapeutic or psychopharmacological antidepressive treatments (for reviews, see Carek et al., 2011; Stoll and Ziemainz, 2012). Despite the impressive effects of exercise on depression among patients with acute major depression, the potential of aerobic exercise as a means of depression prevention is far less clear. Moreover, the frequency, duration, and intensity of exercise that is required to protect an individual from depressive symptoms in the short and long run continues to be debated (Larun, 1996; Dunn et al., 2005; Harvey et al., 2018). Regular moderate- to high-intensity, vigorous aerobic exercise during adolescence and moderate- or even low-intensity regular aerobic exercise (e.g., 3 METs) during adulthood have been found to be particularly effective in this regard (Harvey et al., 2018). According to recent meta-meta-analytic findings (Rebar et al., 2015), low- to moderate-intensity aerobic exercise has moderate effects on the severity of depressive symptoms among non-clinical populations (age > 18 years) after supervised or unsupervised training. This protective effect of even low-intensity aerobic exercise may be attributable to the exercise-induced release of the neurotrophic growth factors that are responsible for nerve growth and synaptic plasticity in the brain, particularly in the brain regions that display significant changes in neural activity and structural changes during depression (e.g., hippocampus; for an overview, see Cotman and Engesser-Cesar, 2002; Carek et al., 2011).

#### Stress Reactivity and Subjective Stress Perceptions

Cognitive, affective, and bodily-related physiological processes are interlinked in the brain. Further, aerobic exercise influences bodily and brain functions (as mentioned earlier). Therefore, one would expect a relationship between aerobic exercise and stress. Influential theoretical models such as the cross-stressor adaptation hypothesis (Sothmann et al., 1996) propose that acute aerobic exercise poses a physical stressor to the body and brain, which, when recurrent, results in an adaptation of the body’s stress response. Since the body’s stress system is also attuned to respond to psychological stressors, it is expected that aerobic exercise will result in cross-stressor tolerance and, consequently, act as a buffer against stress in general, irrespective of whether caused by physical or psychological factors. Laboratory experiments that have (a) explored changes in the psychophysiological indicators of the body’s stress response to acute psychological stressors during and after aerobic exercise and (b) compared physically active individuals and inactive controls have demonstrated empirical support for the cross-stressor adaptation hypothesis (e.g., Childs and de Wit, 2014; for an overview, see Gerber and Pühse, 2009). A few weeks of moderate-intensity aerobic exercise has been linked to better cardiovascular recovery from psychological stress among healthy men and women (Gerber and Pühse, 2009).

Currently, little is known about changes in subjective stress perception. Similar to mental health domains (e.g., mood, depression, anxiety), it is neither clear nor certain whether exercise-induced physiological and psychological adaptation to stress are causally related among healthy individuals. In other words, it is not clear whether exercise-induced physical adaptation to stress is a prerequisite for improvements in mental health and well-being among healthy people. In addition, it is unclear whether the effects of exercise on mental health and well-being are specific to aerobic exercise.

Even though the aforementioned meta-analytic findings have reported moderate effect sizes, past studies that have examined dose-effect relationships have often yielded inconsistent findings that vary depending on the intensity, type, and duration of exercise. With regard to the subjective stress perception, many different types of exercises can reduce the level of perceived stress among healthy individuals who experience moderate to high levels of stress. Beneficial effects have been reported for short- and long-term aerobic exercise, anaerobic exercise, metabolically less-demanding activities (e.g., yoga, relaxation, somatic awareness training), and even a combination of different exercise types (e.g., Neves et al., 2014; Stults-Kolehmainen and Sinha, 2014). The effects appear to be independent of the overall daily physical activity behavior of individuals (e.g., Norris et al., 1992; Magalhaes, 2016). This suggests that regular physical activity and exercise can alter subjective stress perception, irrespective of one’s psychophysiological stress adaptation.

From a psychological perspective, different explanations have been offered to account for the short-term effects of exercise on mental health and well-being during and after engagement in exercise. The factors to which these effects have been attributed range from time outs (i.e., duration of time for which an individual is not preoccupied with stressors, anxiety-inducing factors, and worries) to improvements in self-efficacy and physical self-concept (including body image) and a reduction in body dissatisfaction. Further, there are gender differences in body dissatisfaction. In particular, women tend to be more dissatisfied with their bodies (in terms of size, shape, and weight), compared to men (Fiske et al., 2014; Karazsia et al., 2017). Moreover, body dissatisfaction is a major risk factor for the development of eating disorders, particularly in women (Stice and Shaw, 2002), including female university students (e.g., Herbert et al., 2013). Relatedly, there is evidence to suggest that women are especially motivated to participate in regular exercise programs as a result of their higher levels of body dissatisfaction and concerns about their body weight and shape (Kilpatrick et al., 2005).

### Mental Health and Well-Being Among University Students

University students report high levels of perceived stress and cognitive workload. Recent findings suggest that student counseling centers have been witnessing an increasing number of help-seeking students (for an overview, see Brown, 2018). Moreover, according to recent surveys, every fifth university student experiences mental health problems, which he/she is reportedly unable to cope with independently (University Student Mental Health Survey, 2018). Recent surveys conducted among German university students (e.g., TK-Forsa-Survey, 2012) have revealed that one out of five students provide affirmative responses to questions that assess depressive symptoms and depression severity. Although first-year freshmen are particularly vulnerable to stress and stress-induced depressive and anxiety symptoms (e.g., Ackermann and Schumann, 2010; Farrer et al., 2016), stressors are highly prevalent among all groups of students because they continuously experience stress that is caused by regular examinations, fixed deadlines, and the constant need to perform well to increase their likelihood of later academic achievement. Several studies already found positive correlations between stress and illness in university students on the one hand and between perceived stress, anxiety and depression on the other hand (e.g., Tosevski et al., 2010; Farrer et al., 2016). The relationship between stress and mental health, most notably depression, is not specific to students of a particular university or educational system. Instead, this relationship has been reported among students worldwide (e.g., Ibrahim et al., 2013). Thus, principally, any student, irrespective of his/her culture, might be affected. Nonetheless, students with higher levels of perceived stress are at a significantly higher risk for mental disorders and physical illnesses than the average student. Presumably, this may be the case because such students tend to also engage in detrimental and maladaptive health behaviors to cope with stress (e.g., Mahmoud et al., 2012). Stressed students are also less physically active than their less-stressed counterparts (Nguyen-Michel et al., 2006; Mahmoud et al., 2012).

Despite a wealth of options of university sports programs, many if not all students perceive themselves confronted with strict time regimes and highly demanding learning schedules. This makes it difficult for them to seek additional time-consuming exercise options that will allow them to lead a moderately to highly physically active lifestyle over the course of a regular university day or week. International studies have revealed that approximately half of all students do not meet the WHO and ACSM’s exercise recommendations for gaining health benefits (Irwin, 2004). Moreover, there is evidence to suggest that physical activity, exercise, and stress are reciprocally related and that stress, irrespective of whether it is objectively measured or subjectively perceived, dampens exercise behaviors (Stults-Kolehmainen and Sinha, 2014; Magalhaes, 2016). In addition, psychological stress can also significantly and negatively affect exercise and motor performance by impairing working memory, concentration, and motor control and, in the case of vigorous-intensity exercise, increase the risk of injuries (Stults-Kolehmainen and Sinha, 2014).

Taken together, there is an urgent need to develop physical activity and exercise interventions to promote mental health and well-being among university students. The interventions should fit into their daily working schedule and demands and also fulfill the criteria of being evidence-based. It is crucial for exercise programs that are designed for university students to (a) be time-efficient, (b) require minimal effort and entail minimal injury risk, (c) control for exercise type, intensity, and duration, and (d) allow them to exercise, even when they have an overscheduled working day.

### Aims of the Present Series of Pilot Studies

In accordance with the aforementioned findings, recommendations, and objectives, the present series of pilot studies had two major aims: (a) to investigate mental health, well-being and regular physical activity behavior among university students and (b) to explore the potential effects of short-term weekly aerobic exercise interventions on mental health and well-being among university students. Given the discrepancies in the dose-effect relationships and effect sizes that past studies on exercise and physical activity have reported (see section Introduction), a within-subjects pre-post intervention design was used in the present series of studies to (a) examine the relationships between regular physical activity, mental health, and well-being before the exercise intervention and (b) ascertain the health benefits of exercise (i.e., by comparing pre-intervention and post-intervention health indicators). To better understand the effects of aerobic exercise on mental health and well-being among university students, a randomized control design was chosen, in which the aerobic exercise intervention was compared to a waiting list, a cognitive intervention or a motor skills-related exercise (motor coordination). To ascertain the role of exercise type and duration and the context within which exercise is practiced, the following two studies were conducted: the 6 week online pilot study (i.e., participants exercised in their own homes) and the 2 week laboratory pilot study (i.e., participants exercised under controlled laboratory conditions). Moreover, in contradistinction to past surveys, standardized self-report measures were used to assess mental health and well-being in both the studies. Specifically, self-reported depression, anxiety, perceived stress and coping, body dissatisfaction, and quality of life were measured. In addition, cardiovascular fitness served as an objective measure in the laboratory study.

Taken together, the following key questions were investigated:

(1) Are mental health and well-being related to regular physical activity among university students? In particular, do university students report experiencing depressive symptoms, anxiety, and stress? Are these effects correlated with their regular physical activity behavior?
(2) Can short-term aerobic exercise act as a buffer against perceived stress and promote mental health and well-being in university students after 6 weeks of low to moderate regular exercise?
(3) If yes, will the effects be specific to aerobic exercise (i.e., when compared to a cognitive intervention)?
(4) Will exercise-induced changes in mental health and well-being be accompanied by changes in cardiovascular fitness after 2 weeks of regular exercise? Will the effects be specific to aerobic exercise (i.e., when compared to an exercise intervention that involves motor coordination components)?

## Materials and Methods

### Participants, Initial and Final Sample Sizes, Inclusion and Exclusion Criteria, and Dropouts

Participants were recruited by circulating advertisements on the internet and posting them on online university-specific platforms. The advertisements targeted volunteers who were not members of sports programs and athletes who engaged in regular aerobic exercise, received endurance training, or had been participating in sports competitions. The exclusion criteria were as follows: (a) age < 18 years, (b) regular consumption of illegal substances, (c) former or current diagnosis of or reception of treatment for psychiatric or neurological disorders, (d) a history of cardiovascular or respiratory diseases including diabetes, (e) pregnancy, (f) former or current physical impairments that can hamper engagement in even low-intensity exercises (e.g., injuries, physical handicaps). The same inclusion and exclusion criteria were used in the online and laboratory pilot study.

In total, 185 university students (157 women, 28 men; mean age = 22.54 years, *SD* = 2.93) were willing to participate in the studies^1^. A total of 153 university students (127 women; mean age = 23.05 years, *SD* = 3.54) registered for the online study, and 32 university students registered for the laboratory study (30 women, 2 men; mean age = 22.03 years, *SD* = 2.32). All participants provided written informed consent. All of them completed the first set of measurements at time 1 (T1) and completed an online questionnaire, which included questionnaires that assessed mental health, well-being, and engagement in regular physical activity. Subsequently, their responses were screened to identify missing data and verify their eligibility for inclusion in the study sample (i.e., based on the inclusion and exclusion criteria).

#### Online Pilot Study

Among those who registered for the online study (*N* = 153), 19 individuals were excluded either because their data contained missing values or based on the exclusion criteria. Further, 43 participants withdrew their participation after T1 measurements. Therefore, 91 participants were included in the study protocol and randomly allocated to one of three groups. Random allocation was undertaken using a random event generator. Thirty participants were assigned to the exercise intervention group, 30 participants were assigned to the expressive writing group (i.e., cognitive intervention), and 31 participants were assigned to the waiting list control group. Those assigned to the exercise intervention group participated on average in 11.05 of the 12 exercise sessions (range = 8–12, *SD* = 1.18). Further, those assigned to the expressive writing group participated on average in 11.29 of 12 writing sessions (range = 8–12, *SD* = 1.08). There was no statistically significant difference in the number of participated sessions between the two groups (*p* > 0.1).

Only the data of those who finished the intervention (i.e., exercise or writing intervention), completed all the weekly sessions, and participated in measurements taken at time 2 (T2) were included in the final analysis. Consequently, the data of 74 participants (67 women, 7 men) were included in the data analysis. Specifically, 19, 24, and 31 of them had been assigned to the exercise, expressive writing, and (waiting list) control group, respectively.

#### Laboratory Pilot Study

In the laboratory pilot study, 32 university students (30 women, 2 men) were included in the study protocol and randomly allocated to one of the three groups. Because of the skewed gender distribution, the data of the two male participants were excluded from analyses. Therefore, the final all-female sample consisted of 30 university students (exercise: *n* = 10, motor coordination: *n* = 11, waiting list: *n* = 9).

**FIGURE 1:**
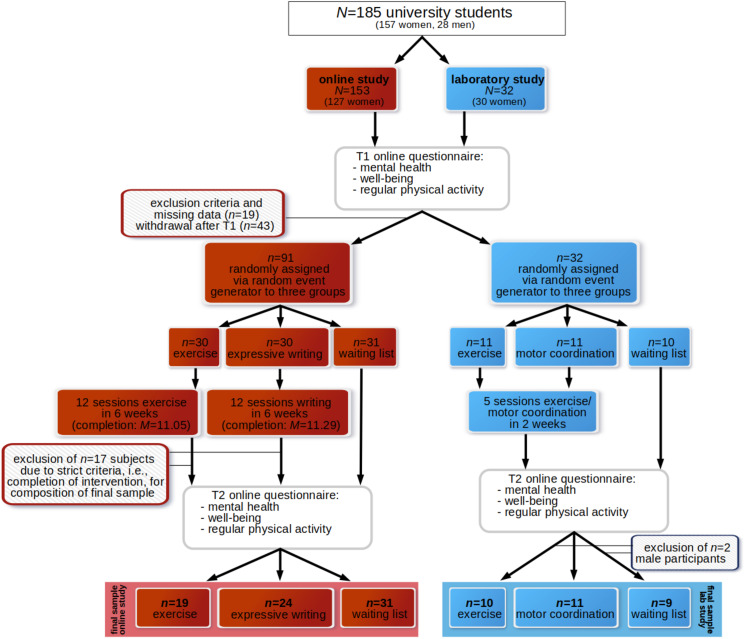
Overview of the study protocol of the randomized controlled trial and the initial and final samples including dropouts.

Figure 1 presents an overview of the protocol of the randomized controlled trial, the initial and final samples included in the pilot studies, and the dropouts.

The participants of the online study were afforded the opportunity to participate in a raffle. They were informed that the winners would win a voucher worth 20 euros, which could be exchanged for things such as sports equipment and communication devices. The participants of the laboratory study were reimbursed individually. They received 50 euros as compensation because they were required to come to the university campus to participate in the exercise intervention.

### Exercise Program

In accordance with past findings (see Introduction), the exercise program focused on aerobic exercise. With regard to the exercise format, exercises were chosen that would allow students to engage in low- to moderate-intensity exercise despite their overscheduled working days. This enhanced the participation of university students who were less intrinsically motivated to engage in regular exercise, could not afford to enroll in fitness or sports courses, and/or wished to exercise without expending too much effort (e.g., at home). Therefore, the exercises were videotaped, and a female and male university student served as the exercise models. In addition, auditory instructions (read by a female instructor) and videos were provided as a part of the supervised exercise training.

**FIGURE 2:**
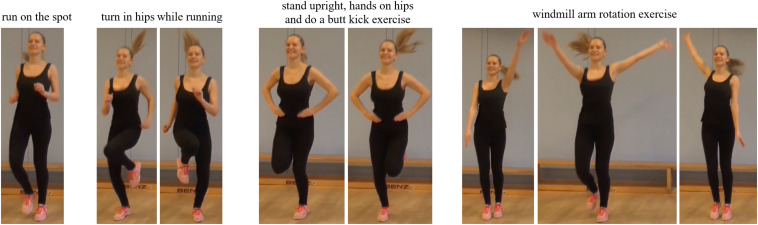
Example of the video-based exercise program (Ex1, Ex2).

Figure 2 depicts an example of the exercises that were included in the supervised video-based exercise program.

Several commercial online exercise and physical fitness programs that promise maximal physical fitness benefits within minimal amounts of time are available to a broad range of users. The aerobic exercises used in this study were designed in accordance with scientific recommendations and definitions. Only exercises that (a) focused on cardiovascular and muscular endurance, (b) required use of key arm and leg muscles for prolonged periods of time, and (c) were related to physical health-related fitness were included (Caspersen et al., 1985). Two different variations of the exercise program were available: Ex1 and Ex2. Tables 1A,B provide an overview of the exercises that were included in Ex1 and Ex2, respectively. Ex1 and Ex2 were provided for three different durations: 8, 12, and 16 min. Ex1 and Ex2 included 1 min warm-up and 1 min cool-down stretching exercises. The intensity of the 16 min aerobic exercises that Ex1 and Ex2 included was piloted using a sample of 10 university students (9 women). Specifically, the effects of the exercise on cardiovascular endurance were assessed using maximal changes in mean heart rate as an empirical estimate (Tanaka et al., 2001). As shown in Table 2, among these ten participants, the aerobic exercises (Ex1 and Ex2) fell within the range for moderate-intensity exercise.

**TABLE 1A:**
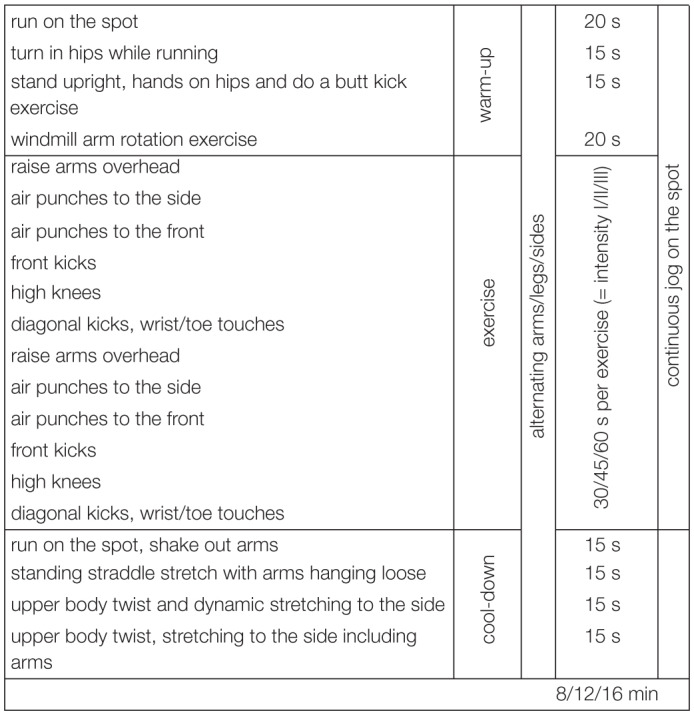
Aerobic exercise, variant 1 (Ex1).

*Exercise 1 (Ex1) comprised warm-up, exercise and cool-down intervals. The duration of each of the 10 exercises determined the total duration of the exercise.*

**TABLE 1B:**
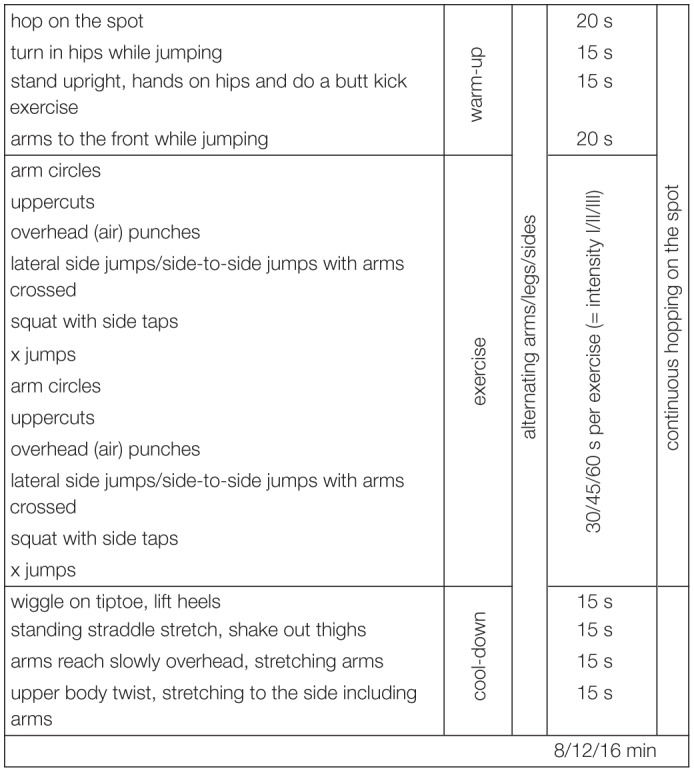
Aerobic exercise, variant 2 (Ex2).

*Exercise 2 (Ex2) comprised warm-up, exercise- and cool-down intervals. The duration of each of the 10 exercises determined the total duration of the exercise.*

**TABLE 2:** Overview of the pilot data investigating the intensity of the aerobic exercise (Ex1) and (Ex2).

age (years)	activity/week (hours)	exercise (Ex1 = 1, Ex2 = 2)	duration (minutes)	age-dependent HRmax	HR-range of moderate activity		meanHR	maxHR	MET-minutes
					Min	Max			
24	5	1	16	191.2	114.72	133.84	124	157	48–64
24	3	2	16	191.2	114.72	133.84	130	167	48–64
28	1	1	16	188.4	113.04	131.88	125	175	48–64
28	3	2	16	188.4	113.04	131.88	135	149	64–128
28	–	1	16	188.4	113.04	131.88	134	171	64–128
28	–	2	16	188.4	113.04	131.88	140	165	64–128
24	4	2	16	191.2	114.72	133.84	143	–	64–128
23	–	2	16	191.9	115.14	134.33	140	–	64–128
27	5	1	16	189.1	113.46	132.37	110	120	48–64
33	4	1	16	184.9	110.94	129.43	122	140	48–64

*16 min duration. HRmax represents age-dependent HRmax values (Tanaka et al., 2001).*

### Procedure and Study Design

As mentioned earlier (see Materials and Methods), the effects of aerobic exercise on mental health and well-being were examined in an online and a laboratory study. In the online pilot study and in the laboratory study, T1 and T2 measurements were compared to ascertain the effects of the exercise intervention. The type and intensity of the aerobic exercises were the same across the two studies.

#### Online Pilot Study

In the online pilot study, the exercise consisted of Ex1 (Table 1A) and Ex2 (Table 1B). The exercise videos were presented online to the participants, so that they could exercise at home. Each participant was required to practice these exercises two times a week for 6 weeks. The intensity of the exercises was increased every 2 weeks, thereby resulting in a total of 12 sessions (i.e., four 8 min sessions, four 12 min sessions, and four 16 min sessions), which entailed engagement in Ex1 or Ex2. With regard to the weekly sessions, the participants always practiced on the same days (i.e., Mondays and Thursdays). They were free to practice either in the morning, afternoon, or evening. They were provided with a link to the website on which the exercise program could be viewed on a computer, tablet, or smartphone. Before each session, an email was sent to the participants to remind them to participate in the session and ensure that they exercise in accordance with the predefined weekly training schedule (see Table 3).

**TABLE 3:** Overview of the experimental design and the exercise schedule of the online pilot study.

T1 measurement	group	online study 6 weeks (2 sessions every week)												T2 measurement
		week 1		week 2		week 3		week 4		week 5		week 6		
		*Mo*	*Th*	*Mo*	*Th*	*Mo*	*Th*	*Mo*	*Th*	*Mo*	*Th*	*Mo*	*Th*	
self-report questionnaire (online)	aerobic exercise: duration (min)	8 min	8 min	8 min	8 min	12 min	12 min	12 min	12 min	16 min	16 min	16 min	16 min	self-report questionnaire (online)
	aerobic exercise: Ex1 vs. Ex2	Ex1	Ex2	Ex1	Ex2	Ex1	Ex2	Ex1	Ex2	Ex1	Ex2	Ex1	Ex2	
	expressive writing	15 min	15 min	15 min	15 min	15 min	15 min	15 min	15 min	15 min	15 min	15 min	15 min	
	control/waiting list	-	-	-	-	-	-	-	-	-	-	-	-	

To evaluate the specificity of the effects of the online exercise program on mental health and well-being, a cognitive intervention (i.e., 6 weeks of expressive writing) was provided to one group of participants (i.e., in addition to the waiting list control group). Expressive writing has been used frequently and successfully in health care programs as a cognitive intervention for stress and emotion regulation. Its efficacy has been demonstrated using different populations, including university students (for an overview, see Baikie and Wilhelm, 2005; Lepore, 2006). In the expressive writing condition, the participants were asked to write about their most stressful weekly event for 15 min, twice a week. Similar to the exercise intervention condition, these participants were also reminded about the weekly writing session, and they were expected to participate in these sessions in accordance with a predefined schedule (i.e., Mondays and Thursdays) for approximately 6 weeks. They were provided with a link to the website on which their personal online diary was hosted. They were also provided with instructions that corresponded to the standard protocol for expressive writing (e.g., Baikie and Wilhelm, 2005). They were asked to write about their most distressing weekly events as expressively as possible, without paying attention to style or grammar, for approximately 15 min. The participants of the waiting list/control group did not receive any instructions. However, they were sent weekly reminders about when the online exercises would begin. Table 3 provides an overview of the training schedule.

#### Laboratory Pilot Study

Similar to the online pilot study, the laboratory pilot study consisted of Ex1 and Ex2. In contradistinction to the online study, the participants of this study were required to come to the laboratory and practice the exercises twice a week across 2 weeks. Similar to the online study, the duration of the exercises was systematically increased across the 2 weeks, thereby resulting in five exercise sessions that comprised one 8 min, two 12 min, and two 16 min exercise sessions, respectively (Table 4).

**TABLE 4:** Overview of the experimental design and the exercise schedule of the laboratory pilot study.

T1 measurement	group	laboratory study (2–3 sessions every week)					T2 measurement
		week 1			week 2		
self-report questionnaire (online), cardiovascular fitness, motor skills	aerobic exercise duration (Ex1 or Ex2)	8 min	12 min	12 min	16 min	16 min	self-report questionnaire (online), cardiovascular fitness, motor skills
	motor coordination	8 min	12 min	12 min	16 min	16 min	
	control/waiting list	-	-	-	-	-	

To examine the specificity of the effects of aerobic exercise on mental health and well-being, three groups were included in the experimental design of the laboratory pilot study (i.e., similar to the online study). The first group received the online aerobic exercise intervention, which has been described in the preceding section. The second group received a motor coordination exercise intervention. In accordance with scientific definitions, this intervention focused on motor skills related to motor coordination and balance rather than aspects related to endurance (Caspersen et al., 1985). An overview of the motor coordination exercise intervention is presented in Table 5. Consistent with the design of the online study, a waiting list control group was included in the laboratory study as well. Weekly exercise sessions were scheduled in such a way that the participants who were assigned to receive the aerobic exercise and motor coordination interventions always visited the laboratory on the same days and at the same time. They were provided with detailed instructions before they began practicing the exercises. The participants of the waiting list/control group did not engage in any exercise, but weekly reminders about the commencement of the interventions were sent to them. An overview of the laboratory training schedule is presented in Table 4.

**TABLE 5:**
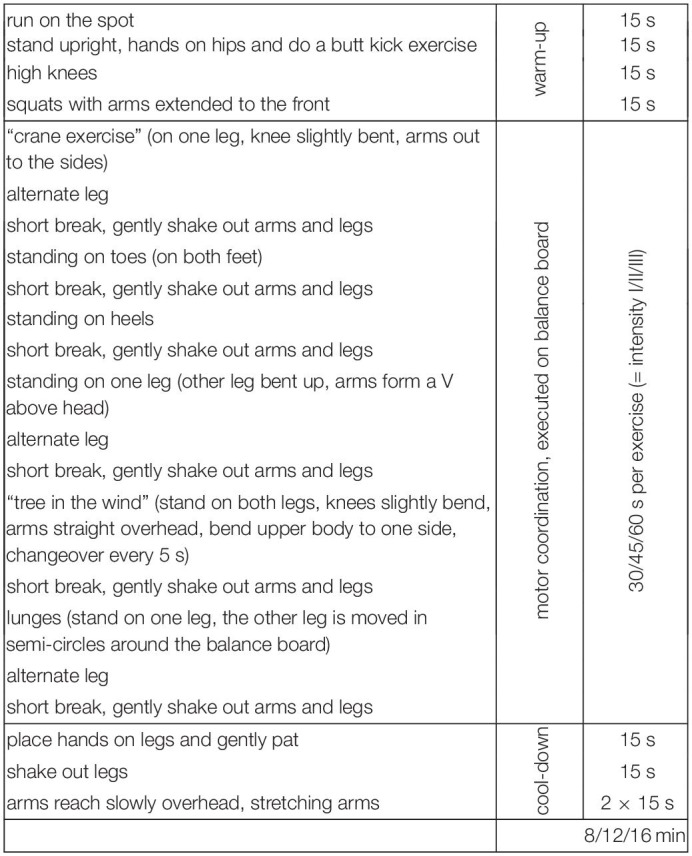
Overview on the motor coordination exercise comprising six exercises of varying duration.

### Measures of Mental Health and Well-Being: Online and Laboratory Pilot Study

The participants of the online and laboratory pilot study received the same standardized self-report measures of mental health and well-being. These self-report assessments were administered at T1 (i.e., before participants were randomly assigned to either the intervention or waiting list groups) and T2 (i.e., at the end of the intervention/waiting period; see Figure 1). In the online study as well as in the laboratory study, T1 measurement was set to 6 weeks after the start of the summer term. Hence, T1 measurement and T2 measurement included a time period in which academic performance can be characterized as high.

Mental health and well-being as well as regular physical activity were assessed using standardized self-report measures of the severity of depressive symptoms, state and trait anxiety, positive and negative affect, stress, quality of life, and physical activity behaviors. The severity of depressive symptoms was assessed using the Beck Depression Inventory (BDI-II; Hautzinger et al., 2010), trait and state anxiety was assessed using the State-Trait Anxiety Inventory (STAI; Spielberger et al., 2001), and positive and negative affect was assessed using the Positive and Negative Affect Schedule (PANAS; Watson et al., 1988). Both the trait and state PANAS scales were used. The state scale assesses positive and negative affect at a given point in time. Using cutoff values, scores of the BDI-II and STAI can be used to classify participants into different groups based on the severity of their depressive symptoms and anxiety levels. Scores of the BDI-II can be classified as follows: no, minimal, mild, and moderate to severe depressive symptoms. The STAI cutoff scores can be used to distinguish between participants with low and high levels of trait and state anxiety. Specifically, cutoff scores of 40 (state) and 44 (trait) can be used to differentiate between healthy adults and those with anxiety disorders (e.g., Julian, 2011). The state subscale of the STAI assesses bodily symptoms that are typically associated with the arousal of the autonomic nervous system (e.g., tension, nervousness, worry, activation). In contrast, the trait subscale assesses “anxiety proneness” and anxiety symptoms in general. Perceived stress and coping strategies were measured using the Stress and Coping Inventory (SCI; Satow, 2012). Its subscales assess the perceived stress that is caused by (a) *uncertainty* (e.g., financial uncertainty, job uncertainty, including university and academic career family and friends), (b) *excessive demands* (i.e., resulting from work, social life), and (c) *the actual experience of negative life events* (e.g., job loss). Subscale and composite scale scores (i.e., sum of subscale scores) can be computed. The SCI also assesses 13 different psychosomatic symptoms of stress such as headache, stomach ache, nightmares, and sexual disinterest. The coping strategies assessed by the SCI can be divided into two types: positive and negative coping strategies. Examples of positive coping strategies are positive thinking, active coping (e.g., trying to address the causes of stress), and social support seeking. Examples of negative coping strategies are alcohol and drug consumption to relieve stress. The WHOQOL-BREF (Whoqol Group, 1998) was used to measure quality of life. The WHOQOL-BREF assesses well-being by measuring quality of life across different life domains, including satisfaction with physical health, well-being (i.e., psychological domain), social relationships, and environmental factors (e.g., financial resources).

Regular physical activity was assessed using the Global Physical Activity Questionnaire (GPAQ, WHO; Armstrong and Bull, 2006). The GPAQ assesses sedentary behaviors and yields scores for three domains of physical activity (i.e., activity at work, travel to and from places, recreational activities). Additionally, body image was assessed using the body dissatisfaction subscale of the Eating Disorder Inventory (EDI-2; Garner, 1991). This assessment measures eating disorder-relevant concerns about body weight and shape.

Table 6 provides an overview of the self-report measures (including their reliability coefficients) that were used in the two pilot studies.

**TABLE 6:** Assessment of mental health and well-being.

concept	type of questionnaire	total no. of items	subscales used in current study (no. of items)	reliability (Cronbach’s alpha) of the questionnaires and subscales used in the online study and the laboratory study
depression	Beck Depression Inventory (BDI-II), German version (Hautzinger et al., 2010)	21	total score (21)	0.84 (*n* = 36 patients with major depression) 0.90 (*n* = 52 patients with major depression leaving hospital) 0.89 (*n* = 315 non-clinical control group) (see Kühner et al., 2007)
anxiety	State-Trait Anxiety Inventory (STAI), German version (Laux et al., 1981)	40	state anxiety (20) trait anxiety (20)	state anxiety 0.91 (*n* = 244 male control group, age range 15–29 years) 0.91 (*n* = 342 female control group, age range 15–29 years) 0.90 (*n* = 171 male students) 0.92 (*n* = 222 female students) trait anxiety 0.89 (*n* = 244 male control group, age range 15–29 years) 0.92 (*n* = 342 female control group, age range 15–29 years) 0.90 (*n* = 171 male students) 0.90 (*n* = 222 female students)
stress	Stress Coping Inventory (SCI) (Satow, 2012)	54	stress overall stress (21) uncertainty (7) excessive demands (7) loss experience (7) psychosomatic symptoms (13) coping positive thinking (4) active stress reduction (4) social support (4) belief/religion (4) alcohol/cigarette consumption (4)	(*n* = 5220 control subjects) 0.82 0.72 0.69 0.69 0.86 0.74 0.74 0.88 0.78 0.75
mood/affect	Positive and Negative Affect Schedule (PANAS) (Watson et al., 1988), German version (Krohne et al., 1996)	20	state positive affect (10)	0.85 (*n* = 349 control subjects)
			state negative affect (10)	0.86 (*n* = 349 control subjects)
			trait positive affect (10)	0.84 (*n* = 480 control subjects)
			trait negative affect (10)	0.86 (*n* = 480 control subjects)
physical activity	Global Physical Activity Questionnaire (GPAQ) (Armstrong and Bull, 2006)	16	physical activity - when working (6) - when traveling (3) - during leisure time (6) sedentary time (1)	retest-reliability (total score) across studies: *r* = 0.58–0.89 (Keating et al., 2019)
quality of life	WHOQOL-BREF (Whoqol Group, 1998)	26	total score (26)	subscales: (*n* = 2073 subjects)
			physical health	0.77
			well-being	0.78
			social relationship	0.82
			environment	0.87
body dissatisfaction	Eating Disorder Inventory (EDI-2), German short version (Thiel and Paul, 1988)	64	body dissatisfaction (9)	0.88 (*n* = 246 patients with anorexia nervosa, *n* = 217 patients with bulimia nervosa)
				0.89 (*n* = 186 female control group)
				0.84 (*n* = 102 male control group)

*Overview of the self-report questionnaires used in the two pilot studies. Reliability indices are reported where available.*

#### Additional Measures: Cardiovascular Fitness, Motivation, and Intervention Effectiveness

At the end of the intervention, the participants of the online study were asked to indicate how motivated they were to continue exercising or writing after the completion of their 6 week interventions. Moreover, they were required to rate the extent to which they believed that exercising or writing had enhanced their well-being and alleviated their stress. Responses were recorded on a 5-point Likert scale that ranged from 1 (not at all) to 5 (absolutely true).

In the laboratory study, the resting heart rate variability (HRV) of each participant (i.e., those assigned to the exercise intervention, motor coordination intervention, and control group) was measured at T1 and T2 to determine the cardiovascular fitness level at T1 and detect exercise-induced changes in cardiovascular fitness (i.e., based on changes in resting HRV) from T1 to T2. They were seated in a comfortable chair with their arms resting on their knees. They were instructed to relax and reduce any cognitive strain during the assessment. Heart rate was measured using a 3-lead electrocardiogram (ECG) with 1000 Hz via a mobile device (BioRadio, Great Lakes NeuroTechnologies)^2^ and recorded for 10 min at rest (i.e., eyes open (EO): 5 min, eyes closed (EC): 5 min), in accordance with an in-house standardized protocol for HRV measurement. To explore the differential effects of the two exercise interventions (aerobic exercise vs. motor coordination), the Beuker-Stemper test (e.g., Stemper, 2016; Bös, 2017) was used. This test battery consists of different exercise tests that measure various aspects of motor performance, including motor coordination, muscle strength, motor speed, and maximal power exercise (e.g., vertical jumps)^3^.

### Data Analysis

The data analytic procedure used in this study is described in the following sections.

#### Mental Health, Well-Being and Relationship With Regular Physical Activity and Cardiovascular Fitness at T1

First, the data of all the participants who had registered for the online study (*N* = 153) and laboratory study (*n* = 30, all-female sample) were analyzed to determine their mental health status and level of well-being and examine the relationship between these variables and the regular physical activity behavior of university students at T1 (i.e., before the commencement of the interventions). Descriptive statistics were computed to ascertain the severity of their depressive and anxiety symptoms, their level of perceived stress, and the extent to which they used different coping strategies, and examine their quality of life, positive and negative affect, body image concerns (body dissatisfaction), and their physical activity behavior. For all the analyses, the data were analyzed based on the gender and the academic degree that the participants were pursuing. Group differences were examined using one-way analysis of variance (ANOVA) (i.e., >2 groups, e.g., academic degree) and independent-samples *t*-test (i.e., 2 groups, e.g., gender). The Wilcoxon rank-sum test (*W*) was used as a non-parametric alternative. When the assumption of the analysis for the ANOVA were not met, the Kruskal-Wallis test (*H*) was used. Partial eta-squared (η*_*p*_*^2^), Cohen’s *d*, and correlation (*r*) coefficients served as measures of effect size. Second, the relationships between self-reported physical activity and depression, anxiety, positive and negative affect, perceived stress, coping, and body dissatisfaction (i.e., indicators of mental health and well-being) at T1 were examined by conducting Pearson’s correlation analyses (two-tailed). Because the sample size was small, data obtained from the participants of the laboratory study were analyzed using Spearman’s rho. In addition, in the laboratory study, Spearman’s correlation coefficients (two-tailed) were computed to examine the relationships between cardiovascular fitness (mean resting HRV), mental health, and well-being at T1.

### Effects of Aerobic Exercise on Mental Health and Well-Being

Next, the effects of the aerobic exercise intervention on self-reported depressive symptoms, anxiety symptoms, positive and negative affect, perceived stress, coping strategies, quality of life, and body dissatisfaction were examined. Effects were analyzed pre to post (T1-T2) exercise and in comparison to expressive writing (online pilot study) or motor coordination (laboratory study), or the control group (waiting list; online pilot study and laboratory pilot study).

With regard to the online pilot study, pre-post and group comparisons (exercise intervention, expressive writing intervention, control group) were undertaken and the interaction between the two factors, “time” (T1 vs. T2) and “group,” was examined using mixed-design ANOVA. Group served as the between-subjects factor, and time served as the within-subjects factor. When the assumptions of ANOVA were violated, a robust analysis for mixed designs, which has been described by Wilcox (2005), was used. Independent- and dependent-samples *t*-tests (parametric and non-parametric alternatives) were used to undertake pairwise comparisons, including the data of the final sample (*n* = 74). All *p*-values reported in this article are uncorrected values, unless otherwise specified. Bonferroni correction was applied for multiple comparisons of *post hoc* tests of the ANOVAs. The samples used in the laboratory pilot study were small. Therefore, pre-post and group comparisons (i.e., exercise intervention, motor coordination intervention, and waiting list control group) were examined using only non-parametric tests (i.e., for independent and dependent samples), namely, the Mann-Whitney *U* test and Wilcoxon test (*Z*). Partial eta-squared (η*_*p*_*^2^) and Cohen’s *d* values served as measures of effect size. All variables were checked for normality prior to statistical analysis.

All statistical analyses were conducted using SPSS (IBM), the software package “R,” and Statistica (statsoft.com).

## Results

Descriptive statistics for the T1 measurements of the initial sample of the online (*N* = 153) and laboratory (*N* = 30; all-female sample) pilot study and the final sample of the online study (*N* = 74) are summarized in Tables 7, 8.

**TABLE 7:** Assessment of mental health and well-being at T1.

		age (years)	depression BDI (0–63)	anxiety state STAI state (20–80)	anxiety trait STAI trait (20–80)	quality of life WHOQOL (%)	body dissatisfaction EDI, female (0–40)	body dissatisfaction EDI, male (0–40)	sedentary time GPAQ (min/week)	regular physical activity (work, travel, leisure) GPAQ (min/week)
online	initial sample at T1 (*n* = 153)	23.05 (3.54) (*n* = 153)	7.73 (6.51) (*n* = 153)	39.59 (9.01) (*n* = 153)	39.33 (10.18) (*n* = 153*)*	67.61 (9.70) (*n* = 147*)*	29.92 (9.52) (*n* = 118)	21.13 (6.68) (*n* = 24)	3129 (1114.40) (*n* = 139)	765.70 (623.59) (*n* = 141)
	final sample at T1 (*n* = 74)	22.84 (4.10) (*n* = 74)	7.18 (6.35) (*n* = 74)	39.59 (9.01) (*n* = 74)	39.31 (9.86) (*n* = 74)	74.75 (10.35) (*n* = 74)	30.21 (9.18) (*n* = 67)	17.14 (5.55) (*n* = 7)	3102 (1157.20) (*n* = 74)	697.50 (720.22) (*n* = 74)
laboratory	final (all-female) sample at T1 (*n* =30)	22.10 (2.33) (*n* = 30)	4.33 (3.86) (*n* = 30)	35.87 (6.12) (*n* =30)	38.97 (8.22) (*n* = 30)	75.91 (9.08) (*n* = 30)	30.17 (9.37) (n =30)	–	3208.33 (1324.60) (*n* = 30)	500.20 (323.92) (*n* = 30)

		**overall stress SCI: sum score uncertainty, demands, loss**	**uncertainty SCI (7–49)**	**excessive demands SCI (7–49)**	**loss experience SCI (7–49)**	**psychosomatic symptoms SCI (13–52)**	**coping: positive thinking SCI (4–16)**	**coping: active stress reduction SCI (4–16)**	**coping: social support SCI (4–16)**	**coping: alcohol/cigarettes consumption SCI (4–16)**

online	initial sample at T1 (*n* = 153)	50.41 (14.93) (*n* = 153)	21.22 (7.40) (*n* = 153)	19.21 (6.46) (*n* = 153)	9.98 (3.85) (*n* = 153)	23.52 (6.47) (*n* = 153)	11.19 (2.07) (*n* = 153)	10.44 (2.74) (*n* = 153)	13.81 (2.25) (*n* = 153)	6.12 (2.51) (*n* = 153)
	final sample at T1 (*n* = 74)	50.54 (13.95) (*n* = 74)	21.66 (7.23) (*n* = 74)	19.43 (5.51) (*n* = 74)	9.45 (3.59) (*n* = 74)	23.72 (6.27) (*n* = 74)	10.95 (1.98) (*n* = 74)	10.55 (2.70) (*n* = 74)	13.86 (2.01) (*n* = 74)	5.81 (2.47) (*n* = 74)
laboratory	final (all-female) sample at T1 (*n* = 30)	43.00 (10.19) (*n* = 30)	18.27 (5.34) (*n* = 30)	16.97 (5.01) (*n* = 30)	7.77 (1.52) (*n* = 30)	21.37 (4.51) (*n* = 30)	10.87 (2.03) (*n* = 30)	10.67 (2.48) (*n* = 30)	14.30 (2.15) (*n* = 30)	5.73 (2.02) (*n* = 30)

*Group means and standard deviations (SD in brackets) are provided for the initial sample (online study), and the final samples taking part in the online pilot study (upper rows), and the laboratory study (lower rows). Scores are provided for T1 measurement. Sample description at T1 includes age and self-report measures. Self-report measures include self-reported depression (BDI, sum score), anxiety (STAI, state and trait sum scores), body dissatisfaction (EDI – sum score, body dissatisfaction scale), perceived stress and coping (SCI, sum scores include overall stress, subscales and coping strategies), quality of life (WHOQOL-BREF, includes overall quality of life, satisfaction with physical health, well-being, social relations and environment), positive and negative affect (trait and state), and physical activity (GPAQ). The range of the scores for the questionnaires and subscales is reported in brackets.*

**TABLE 8:** Assessment of mental health and well-being at T1.

		quality of life satisfaction health WHOQOL (%)	quality of life well-being WHOQOL (%)	quality of life social relations WHOQOL (%)	quality of life environment factors WHOQOL (%)	affect positive trait PANAS (1-5)	affect negative trait PANAS (1-5)	affect positive state PANAS (1-5)	affect negative state PANAS (1–5)
online	initial sample at T1 (*n* =153)	78.30 (12.71) (*n* = 147)	69.42 (14.91) (*n* = 147)	71.88 (19.56) (*n* = 147)	77.55 (12.34) (*n* = 147)	3.22 (0.66) (*n* = 153)	1.83 (0.62) (*n* = 153)	2.82 (0.68) (*n* = 153)	1.41 (0.48) (*n* = 153)
	final sample at T1 (*n* = 74)	78.52 (11.89) (n =74)	70.21 (14.49) (*n* = 74)	72.63 (18.17) (*n* = 74)	77.62 (11.27) (*n* = 74)	3.22 (0.62) (*n* = 74)	1.79 (0.58) (*n* = 74)	2.87 (0.64) (*n* = 74)	1.36 (0.44) (*n* = 74)
laboratory	final (all-female) sample at T1 *(n* = 30)	82.02 (9.45) (*n* = 30)	71.53 (11.27) (*n* = 30)	70.28 (16.48) (*n* = 30)	79.79 (10.21) (*n* =30)	2.39 (0.38) (*n* = 30)	2.48 (0.46) (*n* = 30)	2.82 (0.73) (*n* = 30)	1.24 (0.27) (*n* = 30)

*Group means and standard deviations (SD in brackets) are provided for the initial sample (online study), and the final samples taking part in the online pilot study (upper rows), and the laboratory study (lower rows). Scores are provided for T1 measurement. Sample description at T1 includes age and self-report measures. Self-report measures include self-reported depression (BDI, sum score), anxiety (STAI, state and trait sum scores), body dissatisfaction (EDI – sum score, body dissatisfaction scale), perceived stress and coping (SCI, sum scores include overall stress, subscales and coping strategies), quality of life (WHOQOL-BREF, includes overall quality of life, satisfaction with physical health, well-being, social relations and environment), positive and negative affect (trait and state), and physical activity (GPAQ). The range of the scores for the questionnaires and subscales is reported in brackets.*

### Online Pilot Study (Initial Sample: *n* = 153)

#### Mental Health, Well-Being, and Regular Physical Activity at T1

##### Depression, anxiety, and affect

Among the participants of the initial sample, scores on the BDI-II (*N* = 153) ranged from 0 to 36 (i.e., from no depressive symptoms to severe/major depressive symptoms), and the mean was 7.73 (*SD* = 6.51, median = 6.00). Further, 97 (63.40%) participants obtained scores that ranged from 0 to 8 (i.e., no depressive symptoms), 26 (16.99%) of them reported minimal depressive symptoms (range = 9–13), 22 (14.38%) of them reported mild depressive symptoms (range = 14–19), 7 (4.58%) of them reported moderate depressive symptoms (range = 20–28), and 1 (0.65%) participant reported severe/major depressive symptoms (>29). The severity of depressive symptoms was unrelated to the academic degrees that they were pursuing (bachelor’s vs. master’s vs. “staatsexamen” vs. “other” degrees), *H*(3) = 0.80, *p* > 0.8, η*_*p*_*^2^ = 0.015. There was no significant gender difference (women: *n* = 127, *M* = 7.77, *SD* = 6.63; men: *n* = 26, *M* = 7.54, *SD* = 6.00), *W* = 1650.5, *p* = 1, *r* = 0.

**FIGURE 3:**
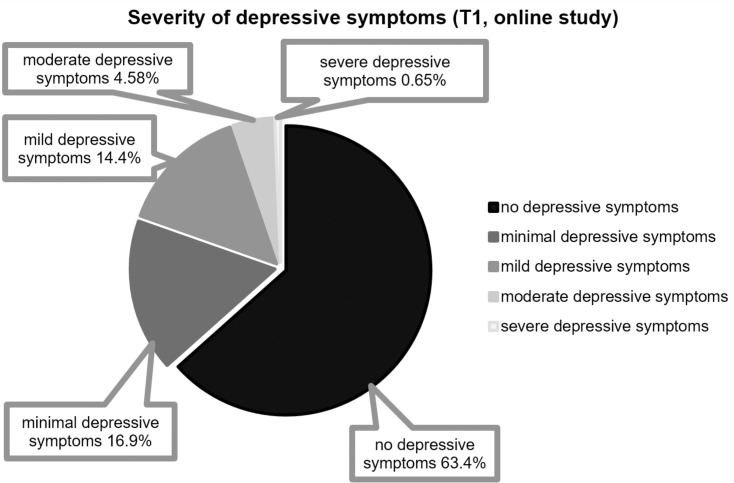
Distribution of the severity of depressive symptoms (BDI-II) (online pilot study; T1; *n* = 153): 0–8 (no depressive symptoms), 9–13 (minimal depressive symptoms, 14-19 (mild depressive symptoms), 20–28 (moderate depressive symptoms), >29 (severe/major depressive symptoms).

Figure 3 provides an overview of the severity of the depressive symptoms (in percentages) reported by the participants of the initial sample at T1.

As shown in Table 7, scores on the trait anxiety subscale of the STAI (*n* = 153) ranged from 20 to 69 (*M* = 39.33, *SD* = 10.18, median = 38.00), and scores on the state anxiety subscale (*n* = 153) ranged from 22 to 65 (*M* = 39.59, *SD* = 9.01, median = 39.00). With regard to trait anxiety, 108 (70.59%) participants obtained scores that lay below the cutoff score that is used to detect clinically relevant symptoms (see section Measures of Mental Health and Well-Being: Online and Laboratory Pilot Study), and 45 (29.41%) of them scored above the clinical cutoff score. Moreover, 89 (58.17%) participants obtained low scores on the state anxiety subscale, and 64 (41.83%) of them obtained high scores on the state anxiety subscale. The severity of their trait, *F*(3, 149) = 0.60, *p* = 0.613, η*_*p*_*^2^ = 0.012, and state anxiety, *F*(3, 149) = 1.18, *p* = 0.32, η*_*p*_*^2^ = 0.023, was unrelated to the academic degree that they were pursuing. Gender differences in state (women: *n* = 127, *M* = 39.28, *SD* = 8.90; men: *n* = 26, *M* = 41.08, *SD* = 9.56), *W* = 1474, *p* = 0.391, *r* = −0.07, and trait anxiety (women: *n* = 127, *M* = 39.32, *SD* = 10.40; men: *n* = 26, *M* = 39.42, *SD* = 9.27), *W* = 1613, *p* = 0.855, *r* = −0.02, were not significant.

The initial sample (*N* = 153) obtained mean scores that ranged from 1.20 to 4.80 (*M* = 2.82, *SD* = 0.68, median = 2.80), and from 1.30 to 4.80 (*M* = 3.22, *SD* = 0.66, median = 3.20) on the measures that were used to assess state and trait positive affect (PANAS), respectively. When compared to positive affect (Table 8), the participants reported lower levels of state (range = 1.00–3.30, *M* = 1.41, *SD* = 0.48, median = 1.20) and trait negative affect (range = 1.00–4.30, *M* = 1.83, *SD* = 0.62, median = 1.70). Positive affect (state and trait) was unrelated to the academic degrees that they were pursuing, state positive: *F*(3, 149) = 0.78, *p* = 0.51, η*_*p*_*^2^ = 0.015; trait positive: *F*(3, 149) = 0.10, *p* = 0.96, η*_*p*_*^2^ = 0.002. The academic degrees that they were pursuing had no effect on their state, *H*(3) = 0.51, *p* = 0.91, η*_*p*_*^2^ = 0.017, or trait negative affect, *F*(3, 149) = 0.72, *p* = 0.54, η*_*p*_*^2^ = 0.014. Gender differences in state (women: *n* = 127, *M* = 2.84, *SD* = 0.65; men: *n* = 26, *M* = 2.72, *SD* = 0.80), *t*(151) = 0.82, *p* = 0.416, *d* = 0.18, and trait positive affect (women: *n* = 127, *M* = 3.25, *SD* = 0.66; men: *n* = 26, *M* = 3.04, *SD* = 0.63), *t*(151) = 1.50, *p* = 0.136, *d* = 0.32, and state (women: *n* = 127, *M* = 1.40, *SD* = 0.47; men: *n* = 26, *M* = 1.47, *SD* = 0.51), *W* = 1431.5, *p* = 0.282, *r* = −0.09, and trait negative affect (women: *n* = 127, *M* = 1.82, *SD* = 0.60; men: *n* = 26, *M* = 1.87, *SD* = 0.72), *W* = 1628, *p* = 0.913, *r* = −0.01, were not significant.

#### Perceived Stress and Coping

As can be inferred from Table 8, the scores that the participants (*N* = 153) of the initial sample obtained on the SCI revealed that they had been experiencing stress due to uncertainty (e.g., financial uncertainty, job uncertainty, and uncertainty about other domains of life such as academic performance, career, family, and friends) and excessive demands (e.g., work, social life). Perceived stress due to uncertainty and excessive demands was greater than perceived stress due to actual experiences of negative life events (e.g., job loss) within this sample (uncertainty*: M* = 21.22, *SD* = 7.40; excessive demands: *M* = 19.21, *SD* = 6.46; loss experience: *M* = 9.98, *SD* = 3.85). Women and men did not differ in the amount of overall reported stress symptoms (global scores on the SCI: women: *n* = 127, *M* = 50.80, *SD* = 14.83; men: *n* = 26, *M* = 48.50, *SD* = 15.55), *t*(151) = 0.72, *p* = 0.475, *d* = 0.15, and women and men scored equally high in psychosomatic symptoms including different psychosomatic symptoms such as headache, stomachache, nightmares, sexual disinterest (women: *n* = 127, *M* = 23.84, *SD* = 6.42; men: *n* = 26, *M* = 21.96, *SD* = 6.61), *W* = 1930, *p* = 0.176, *r* = 0.11. Positive coping strategies included positive thinking, active coping (e.g., trying to resolve the causes of stress) or social support seeking. Negative coping strategies included alcohol and drug consumption for stress relaxation. Overall, positive coping strategies were reported more often than negative coping strategies (see Table 8). Social support seeking emerged as the most frequently used positive coping strategy. Notably, female participants tended to use this positive coping strategy significantly more frequently than male participants did (women: *n* = 127, *M* = 14.07, *SD* = 2.12; men: *n* = 26, *M* = 12.54, *SD* = 2.50), *W* = 2283, *p* < 0.01, *r* = 0.25. Alcohol consumption and cigarette smoking, which are negative coping strategies, were more frequently reported by male students than by female students (women: *n* = 127, *M* = 5.92, *SD* = 2.59; men: *n* = 26, *M* = 7.12, *SD* = 1.86), *W* = 989.5, *p* < 0.001, *r* = −0.27. The academic degrees that they were pursuing did not have a significant effect on overall perceived stress, *F*(3, 149) = 1.06, *p* = 0.368, η*_*p*_*^2^ = 0.021, or psychosomatic symptoms, *F*(3, 149) = 0.13, *p* = 0.940, η*_*p*_*^2^ = 0.003. With regard to positive coping strategies, the academic degrees that the participants were pursuing had no effect on positive thinking, *F*(3, 149) = 0.75, *p* = 0.524, η*_*p*_*^2^ = 0.015, and social support seeking, *H*(3) = 2.00, *p* = 0.572, η*_*p*_*^2^ = 0.007. However, with regard to active stress reduction, there were differences between the groups that were pursuing different academic degrees, *H*(3) = 8.25, *p* < 0.05, η*_*p*_*^2^ = 0.035. Specifically, the observed difference was significantly greater among students who were pursuing a master’s degree than among those who were pursuing a bachelor’s degree (bachelor’s degree: *M* = 9.96, *SD* = 2.60; master’s degree: *M* = 11.64, *SD* = 3.23). No significant difference emerged for alcohol consumption, which is a negative coping strategy, *H*(3) = 5.85, *p* = 0.119, η*_*p*_*^2^ = 0.017.

#### Quality of Life

It can be inferred from Table 8 that there were differences between the four major domains of self-reported quality of life (WHOQOL-BREF; *n* = 147). The participants were reportedly more satisfied with their environment (e.g., financial resources, health, and social care: *M* = 77.55, *SD* = 12.34), and their physical health (*M* = 78.30, *SD* = 12.71) than with their level of well-being (psychological domain: *M* = 69.42, *SD* = 14.91) and social relationships (*M* = 71.88, *SD* = 19.56), *F*(3, 438) = 20.12, *p* < 0.001, η*_*p*_*^2^ = 0.12. This pattern did not differ between the groups pursuing different academic degrees or between the female and male participants, all *ps* > 0.1.

#### Body Dissatisfaction

On the assessment that was used to assess body dissatisfaction (EDI-2), the participants (*n* = 142) obtained a mean score of 28.44 (*SD* = 9.67). Further, consistent with the literature (also see Table 7), female university students obtained significantly higher scores than their male counterparts (women: *M* = 29.92, *SD* = 9.52; men: *M* = 21.13, *SD* = 6.68), *t*(140) = 4.31, *p* < 0.001, *d* = 0.97.

#### Regular Physical Activity

Table 7 presents descriptive statistics (*n* = 141) for composite scores (i.e., physical activity across the three domains of daily life, namely, activity at work, travel to and from places, and recreational activities) on the GPAQ, which is based on the recommendations of the WHO. Notably, their time spent sitting was higher than the recommended duration. Their average time spent sitting was reportedly 7.45 h/day, and this amounted to an average of 3129 min/week (*SD* = 1114.40). The mean durations for which they engaged in physical activity (i.e., minutes per week) were as follows: moderate-intensity activity at work = 160.40 min/week, vigorous-intensity activity at work = 38.44 min/week, travel to and from places = 239.78 min/week, moderate-intensity recreational activities = 158.60 min/week, and vigorous-intensity recreational activities during leisure time = 168.00 min/week. These durations amounted to a total of 765.70 min/week (*SD* = 623.59). Further, 124 (87.32%) participants did not engage in vigorous-intensity activity at work, and 78 (54.93%) participants did not engage in moderate-intensity activity at work. Additionally, 34 (23.94%) and 38 (26.76%) participants did not engage in vigorous- and moderate-intensity activity during their leisure time, respectively. With regard to the total duration for which they engaged in physical activity on a weekly basis (i.e., including activity at work, during transport, and leisure time), 21 (14.79%) participants did not meet the WHO criterion of 150 min of moderate-intensity physical activity, and 7 (4.93%) of them did not meet the WHO criterion of 75 min of vigorous-intensity physical activity. The participants achieved less than 600 MET-minutes per week. Gender differences in sedentary behaviors (i.e., per week) were not significant (women: *M* = 3108.12, *SD* = 1020.55; men: *M* = 3232.17, *SD* = 1527.17), *W* = 1317.5, *p* = 0.927, *r* = −0.01. The same was found for overall physical activity per week (women: *M* = 753.80, *SD* = 606.48; men: *M* = 824, *SD* = 712.51), *W* = 1316, *p* = 0.631, *r* = −0.04. Moreover, overall physical activity and sedentary time did not differ across the groups that differed in the academic degrees that they were pursuing, overall physical activity: *H*(3) = 3.17, *p* = 0.366, η*_*p*_*^2^ = 0.008; sedentary time: *H*(3) = 5.03, *p* = 0.170, η*_*p*_*^2^ = 0.002.

#### Relationships Between Regular Physical Activity, Mental Health, and Well-Being

Correlation analysis revealed that there were significant relationships between overall physical activity (GPAQ) and self-reported depression, *r* = −0.22, *p* < 0.05. Overall physical activity was also negatively correlated with state, *r* = −0.27, *p* < 0.001, and trait anxiety, *r* = −0.26, *p* < 0.001, and body dissatisfaction, *r* = −0.21, *p* < 0.05. It was also positively correlated with positive affect, trait: *r* = 0.28, *p* < 0.001; state: *r* = 0.30, *p* < 0.005. A significant negative correlation emerged between overall physical activity and psychosomatic stress symptoms, *r* = −0.21, *p* < 0.01. Overall physical activity was positively correlated with quality of life, sum score: *r* = 0.27, *p* < 0.001, the different domains across which it was measured, namely, physical health, *r* = 0.20, *p* < 0.001, well-being, psychological domain: *r* = 0.31, *p* < 0.005, and social relationships, *r* = 0.17, *p* < 0.05, and coping, *r* = 0.17, *p* < 0.05, and marginally with support seeking, *r* = 0.16, *p* = 0.059. These correlations emerged among T1 measurements, which were collected using the following standardized assessments: the BDI-II, STAI (trait and state anxiety), EDI-2 (body dissatisfaction), WHOQOL-BREF, SCI, and PANAS (trait and state affect).

### Short-Term Aerobic Exercise, Mental Health, and Well-Being (Online Study; *n* = 74, Final Sample)

#### Exercise, Depression, Anxiety, and Affect

**FIGURE 4:**
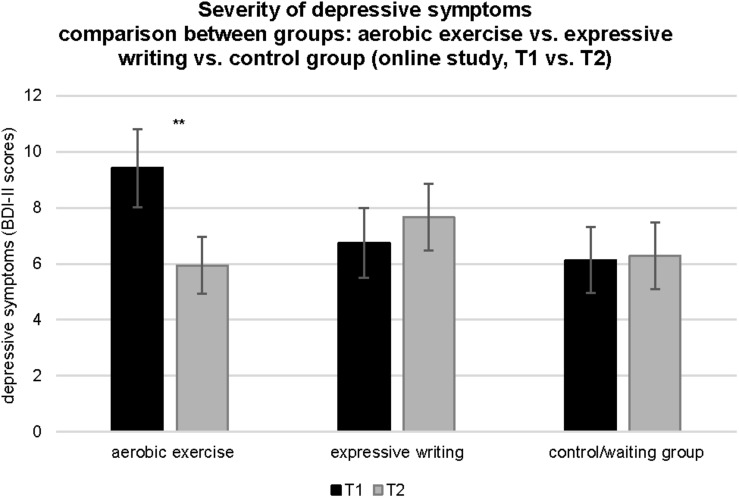
Comparison of BDI-II scores across time (T1 vs. T2) and between experimental conditions (online pilot study: aerobic exercise group, expressive writing group, control group/waiting list). Vertical bars denote ± standard errors. Significant differences between T1 and T2 are indicated (*p ≤ 0.05; **p ≤ 0.01; ***p ≤ 0.001).

There was a significant interaction between time and group for depression, *F*(2, 71) = 5.23, *p* < 0.005, η*_*p*_*^2^ = 0.13. Specifically, there was a decrease in self-reported depressive symptoms between T1 and T2 among the participants assigned to the exercise intervention group, *t*(18) = 3.38, *p* < 0.005, *d* = 0.62. As shown in Figure 4, at T1, the participants of the aerobic exercise group scored on average two scores higher on the BDI-II than the participants of the expressive writing group or the participants of the waiting list control group. This difference was not statistically different, *F*(1, 72) = 1.70, *p* > 0.1, η*_*p*_*^2^ = 0.04. Comparisons of descriptive statistics revealed that, among those assigned to the aerobic exercise intervention group, 73.68% demonstrated improvements in depressive symptoms between T1 and T2. Among those assigned to the expressive writing intervention group, 45.8% demonstrated improvements in depressive symptoms between T1 and T2. In contrast, only 36.6% of the waiting list control participants demonstrated improvements in depressive symptoms between T1 and T2. The difference scores (T1 vs. T2) were significantly different between the three groups, *F*(2, 71) = 5.23, *p* ≤ 0.01, η*_*p*_*^2^ = 0.13.

*Time* had a main effect on state anxiety, *F*(1, 71) = 14.16, *p* < 0.001, η*_*p*_*^2^ = 0.06. Specifically, there was an increase in state anxiety between T1 and T2. The interaction between *time* and *group* was marginally significant, *F*(2, 71) = 2.50, *p* = 0.090, η*_*p*_*^2^ = 0.02, as did the main effect of *group*, *F*(2, 71) = 2.45, *p* = 0.094, η*_*p*_*^2^ = 0.04. *Post hoc* tests revealed a marginally significant difference between the exercise and expressive writing intervention groups at T2 (exercise: *M* = 40.90, *SD* = 8.69; expressive writing: *M* = 48.79, *SD* = 12.88), *p* = 0.083. There were no significant effects for trait anxiety. Neither their main effects nor the interaction effect on trait anxiety was significant, *group*: *Q* = 0.89, *p* = 0.423; *time*: *Q* = 0.95, *p* = 0.336; group × time: *Q* = 1.34, *p* = 0.281.

With regard to both positive and negative affect (state), the main effect of *time* was significant, state positive affect: *F*(1, 71) = 5.10, *p* < 0.05, η*_*p*_*^2^ = 0.02; state negative affect: *Q* = 8.83, *p* < 0.01. Positive affect had decreased and negative affect had increased by the end of the 6-week intervention, and this trend did not differ across the three groups. *Time*: *Q* = 5.35, *p* < 0.05 and *group*: *Q* = 4.52, *p* < 0.05 had significant main effects on trait positive and negative affect, respectively.

#### Exercise, Perceived Stress, and Stress Coping

With regard to overall stress perception (perceived stress), there was a significant change in scores across *time*, *F*(1, 71) = 15.80, *p* < 0.001, η*_*p*_*^2^ = 0.04, and the interaction between *time* and *group* was significant, *F*(2, 71) = 5.69, *p* < 0.01, η*_*p*_*^2^ = 0.03. Overall, perceived stress (sum of the scores yielded by the four subscales of the SCI) significantly decreased between T1 and T2, and this trend was particularly pronounced among the exercise intervention group participants, *t*(18) = 4.37, *p* < 0.001, *d* = 0.72. Perceived stress caused by uncertainty significantly increased between T1 and T2 (*time)*, *F*(1, 71) = 23.94, *p* < 0.001, η*_*p*_*^2^ = 0.25. Further, the interaction between *time* and *group, F*(2, 71) = 5.39, *p* < 0.01, η*_*p*_*^2^ = 0.13, was significant. Specifically, there was a reduction in perceived stress due to uncertainty only among the aerobic exercise intervention group participants, *t*(18) = 5.15, *p* < 0.001, *d* = 0.77. *Time* had a significant main effect on stress due to excessive demands, *F*(1, 71) = 4.75, *p* < 0.05, η*_*p*_*^2^ = 0.02, and there was a marginally significant interaction between *time* and *group*, *F*(2, 71) = 2.54, *p* = 0.086, η*_*p*_*^2^ = 0.02. No significant main or interaction effects emerged for psychosomatic stress symptoms, the experience of loss, and coping strategies, all *p*s > 0.05.

#### Exercise and Quality of Life

No significant main effect emerged for any of the quality of life domains, all *p*s > 0.05. Further, none of the interaction effects were significant, all *p*s > 0.05.

#### Exercise and Body Dissatisfaction

*Time* had a significant main effect on body dissatisfaction (EDI-2), *F*(2, 71) = 5.18, *p* < 0.05, η^2^ = 0.004. All the groups demonstrated similar slight decreases in their level of body dissatisfaction over time (i.e., from T1 to T2).

#### Manipulation Check: Motivation and Intervention Effectiveness

**FIGURE 5:**
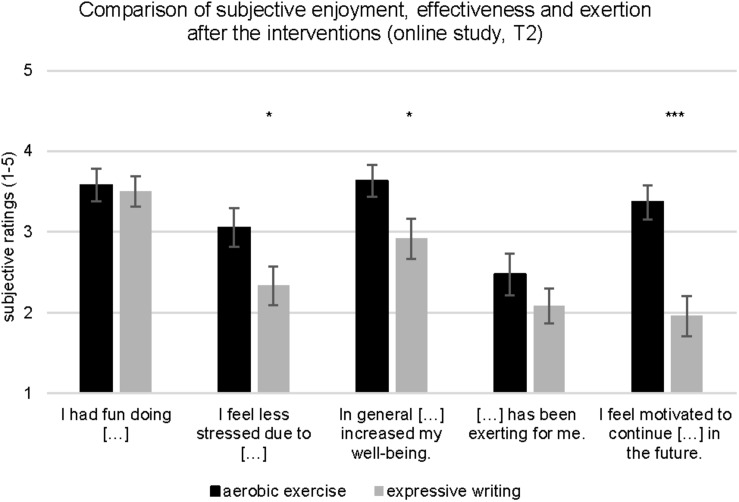
Participants’ ratings of enjoyment, subjective effectiveness of the intervention and exertion (online pilot study: aerobic exercise group, expressive writing group). Vertical bars denote ± standard errors. Significant differences between groups are indicated (*p ≤ 0.05; **p ≤ 0.01; ***p ≤ 0.001).

At T2, the participants of the aerobic exercise and expressive writing intervention groups were required to indicate how motivated they were to continue exercising or writing and rated the effectiveness of the intervention that they had received (i.e., exercising or writing) in enhancing well-being and alleviating perceived stress. The participants of the exercise and the expressive writing intervention groups reported that they enjoyed taking part in the intervention. However, when compared to the participants of the expressive writing intervention group, those who belonged to the aerobic exercise intervention group were significantly more motivated to continue exercising, *t*(41) = 4.10, *p* < 0.001, *d* = 1.23. Moreover, as shown in Figure 5, the extent to which the intervention was perceived to have been effective in alleviating stress, *t*(41) = 2.03, *p* < 0.05, *d* = 0.63, and enhancing well-being, *t*(41) = 2.10, *p* < 0.05, *d* = 0.65, was greater among the exercise intervention group participants than among the expressive writing intervention group participants (Figure 5).

### Laboratory Pilot Study (*n* = 30, All-Female Sample)

#### Mental Health, Well-Being, and Regular Physical Activity at T1

##### Depression, anxiety, affect, perceived stress, body dissatisfaction, and quality of life

It can be inferred from Table 7 that, at T1, self-reported depressive symptoms (BDI-II) were less severe in the sample of the laboratory pilot study than in the online study sample. Specifically, 25 (83.3%) of them had no depressive symptoms, and 5 (16.7%) of them reported minimal symptoms. None of them reported moderate or clinically relevant depressive symptoms (*M* = 4.33, *SD* = 3.86, median = 4.00). At T1, both samples reported similar levels of trait anxiety, but state anxiety levels were lower in the laboratory study sample than in the online study sample (Table 7). In the all-female sample the all-female sample of the laboratory study, 9 (30%) and 7 (23.3%) participants obtained high scores on the assessments that were used to measure state and trait anxiety, respectively. Differences in positive and negative affect, quality of life, and body dissatisfaction between the two samples were not significant (Tables 7, 8). Similar to the results that emerged for the online study sample, the participants of the laboratory study were more stressed because of uncertainty and excessive work demands than because of actual experiences of negative life events (Table 8).

#### Physical Activity, Cardiovascular Fitness, Mental Health, and Well-Being

Similar to the online study sample, the laboratory study sample’s average time spent sitting was 7.6 h/day. The mean durations for which they engaged in physical activity (i.e., per week) were as follows (total: *M* = 500.17 min/week, *SD* = 323.92): activity at work = 57.17 min/week, travel to and from places = 189.20 min/week, and leisure-time activity = 253.80 min/week. Referring to the WHO’s recommended levels of physical activity, 9 (30%) of them engaged in < 150 min of moderate-intensity activity, and 2 (6.7%) of them engaged in < 75 min of vigorous-intensity activity.

Their regular physical activity behavior (GPAQ; calculated as MET-minutes per week) was significantly and negatively correlated with coping strategies, social support seeking: *r* = −0.392, *p* = 0.032, at T1. Cardiovascular fitness [HRV-HF (n.u.), EO] at T1 was significantly and negatively correlated with self-reported state anxiety, *r* = −0.509, *p* < 0.005, and marginally negatively correlated with self-reported depression, *r* = −0.328, *p* = 0.077. Significant negative correlations emerged for negative state affect, *r* = −0.362, *p* < 0.05, and marginally significant positive correlations emerged for one quality of life domain, namely, social relationships, *r* = 0.345, *p* = 0.062.

### Effects of Short-Term Aerobic Exercise on Well-Being

At T1, there was no significant difference between the aerobic exercise intervention, motor coordination intervention, and waiting list control group. Within-group comparisons (T1 vs. T2) of T1 and T2 measurements (i.e., depression, anxiety, perceived stress, affect, quality of life, and coping) yielded a few significant findings. The motor coordination intervention group demonstrated a significant decrease in social support seeking from T1 to T2, *Z* = −2.13, *p* < 0.05, *d* = 1.68. The waiting list control group demonstrated a significant decrease for quality of life related environmental factors, *Z* = −2.08, *p* = < 0.05, *d* = 1.92, and a marginal decrease in state positive affect, *Z* = −1.68, *p* = 0.092, *d* = 1.35, between T1 and T2.

Comparisons between the three groups at T2 showed the following results. At T2, the aerobic exercise and motor coordination intervention groups did not differ significantly from the waiting list control group in the following variables: self-reported depression (aerobic exercise: *M* = 4.20, *SD* = 5.87 vs. waiting list: *M* = 5.22, *SD* = 3.83, *U* = 35.50, *p* > 0.3, *d* = 0.62; motor coordination: *M* = 2.27, *SD* = 4.00 vs. waiting list: *U* = 27.50, *p* = 0.095, *d* = 0.80) and state (aerobic exercise: *M* = 41.00, *SD* = 9.98 vs. waiting list: *M* = 38.22, *SD* = 3.83, *U* = 38.00, *p* > 0.1, *d* = 0.27; motor coordination: *M* = 34.90, *SD* = 6.71 vs. waiting list: *U* = 29.50, *p* > 0.1, *d* = 0.72) and trait anxiety (aerobic exercise: *M* = 38.90, *SD* = 6.14 vs. waiting list: *M* = 38.00, *SD* = 9.31, *U* = 40.50, *p* > 0.1, *d* = 0.17; motor coordination: *M* = 36.36, *SD* = 7.76 vs. waiting list: *U* = 46.00, *p* > 0.1, *d* = 0.12).

Differences between the aerobic exercise and waiting list control group were significant for trait positive affect (aerobic exercise: *M* = 2.47, *SD* = 0.25 vs. waiting list: *M* = 2.09, *SD* = 0.37, *U* = 17.00, *p* < 0.05, *d* = 1.23), marginally significant for state positive affect (aerobic exercise: *M* = 2.94, *SD* = 0.73 vs. waiting list: *M* = 2.38, *SD* = 0.054, *U* = 23.50, *p* = 0.079, *d* = 0.88), and significant for social support seeking (aerobic exercise: *M* = 13.20, *SD* = 2.15 vs. waiting list: *M* = 15.0, *SD* = 1.58, *U* = 23.50, *p* < 0.05, *d* = 1.06). When compared to the waiting list control group, the motor coordination intervention group obtained significantly higher scores on the assessment that was used to assess trait positive affect (motor coordination: *M* = 2.43, *SD* = 0.32), *U* = 23.50, *p* ≤ 0.05, *d* = 0.99, and marginally lower scores on the assessment that was used to measure social support seeking (motor coordination: *M* = 13.64, *SD* = 2.11), *U* = 26.50, *p* = 0.080, *d* = 0.85.

There was no significant change in cardiovascular fitness [resting HRV-HF (n.u.)] between T1 and T2. However, at T2, HRV values [HRV-HF (n.u.), EO] were lower for the participants of the waiting list group than the aerobic exercise and motor coordination intervention groups (waiting list: *M* = 27.40, *SD* = 12.75 vs. aerobic exercise: *M* = 46.10, *SD* = 21.36, *U* = 20.00, *p* ≤ 0.05, *d* = 1.06; waiting list vs. motor coordination: *M* = 46.72, *SD* = 13.50, *U* = 13.00, *p* ≤ 0.005, *d* = 1.58). Similar albeit marginally significant findings emerged for EC HRV-HF (n.u.) measurements (waiting list: *M* = 27.03, *SD* = 22.06 vs. aerobic exercise: *M* = 48.05, *SD* = 24.48, *U* = 24.00, *p* = 0.095, *d* = 0.86; waiting list vs. motor coordination: *M* = 46.38, *SD* = 15.71, *U* = 26.00, *p* = 0.080, *d* = 0.87). Group differences in motor performance were not significant at T2. However, the motor coordination intervention showed significant improvements in motor performance on the balance board (i.e., T1 vs. T2), which was included as subtest in the Beuker-Stemper test battery [number of ground contacts (errors): T1: *M* = 21.16, *SD* = 6.30 vs. T2: *M* = 16.3, *SD* = 3.91, *Z* = 2.05, *p* < 0.05, *d* = 1.57].

## Discussion

University students experience high cognitive workloads throughout the entire duration of their academic programs. Recent surveys have revealed that up to 25% of university students feel so psychologically stressed that they are unable to independently cope; they also experience anxiety and depressive symptoms (e.g., TK-Forsa-Survey, 2012; University Student Mental Health Survey, 2018). In addition, at universities, weekly working schedules follow a strict time regime with daily lectures and courses and exams. Taken together, this can promote sedentary behaviors among university students in the long run. Epidemiological studies have found that physical inactivity and sedentary behavior are major risk factors for mortality and chronic diseases (e.g., Same et al., 2016; Young et al., 2016). The present series of pilot studies examined the relationships between mental health, well-being, and regular physical activity among university students. Furthermore, they examined the extent to which short-term aerobic exercise interventions (i.e., 2–6 weeks of regular engagement in low- to moderate-intensity aerobic exercise) act as a buffer against perceived stress, anxiety, and depression and promote well-being (e.g., affect, quality of life). Mental health and well-being were assessed using self-report measures of the severity of depressive symptoms, state and trait anxiety, positive and negative affect, perceived stress, coping strategies, quality of life, body dissatisfaction, and self-reported regular physical activity. The effectiveness of the intervention was ascertained by comparing T1 and T2 measurements. The samples were undergraduate and graduate students, who were recruited from German universities. They either participated in an online study, which lasted for 6 weeks, or in a laboratory study, which lasted for 2 weeks and required them to visit the laboratory. In the online study as well as in the laboratory study, short-term aerobic exercise comprised low to moderate intensity aerobic exercise and effects were compared to control conditions comprising a cognitive intervention (online study), motor coordination exercise (laboratory study) and waiting list (online study and laboratory study).

### Mental Health and Well-Being Among University Students

#### Online and Laboratory Pilot Study

The present results are consistent with the findings of recent health surveys on mental health and well-being. Overall, 36.6% of the participants of the online study, who were recruited from different universities in Germany, reported minimal and mild (31.3%) or moderate (4.58%) to severe (0.65%) depressive symptoms (i.e., as measured by the BDI-II) at T1 (i.e., before the commencement of the interventions). The BDI-II assesses the severity of depressive symptoms during the past 2 weeks. Typically, women have a higher risk of developing major depressive disorder than men do. In the online study (*N* = 153), the severity of depressive symptoms did not vary as a function of gender or the academic degrees that the participants were pursuing. Further, there were no significant differences in BDI-II scores between the all-female sample of the laboratory study (*N* = 30) and the sample of the online study, which consisted of men and women. In addition, their anxiety scores were comparable to the norms that have been reported for college students and young adults. Nevertheless, at T1, 41.83% of the participants of the initial sample (which included both women and men) scored above the cutoff scores that have previously been reported for state anxiety (e.g., Julian, 2011). Women were overrepresented in the online study sample. This trend is consistent with past observations regarding online studies and research on exercise. Specifically, when compared to men, women have been found to be more willing to participate in online studies (e.g., Smith, 2008), especially those that pertain to exercise, health, and well-being (Kilpatrick et al., 2005).

Past studies on the mental health and well-being of university students have been using samples of students who had contacted the health counseling service providers of their university to seek help for private or psychological problems (e.g., Thees et al., 2012; Heilmann et al., 2015). Many such studies have been conducted using samples of medical students because psychological stress and academic demands are more pronounced among such individuals (Dahlin et al., 2005). Therefore, past findings may be specific to particular groups of students, including those who are already at high risk for stress-related disorders. In comparison, the students who participated in this study were not selected based on the academic degree that they were pursuing or existing health problems. In contrast, only students without a clinical diagnosis of neurological, somatic, and psychiatric disorders were eligible to participate in the present series of pilot studies. As the current findings may be representative of the population of students without chronic health conditions, it is alarming that 36.6% of the participants of the online study (i.e., female and male university students) reported depressive symptoms and that 41.83% of them reportedly had high levels of state anxiety. Recent online surveys on the mental health of university students have reported similarly high rates (see section Introduction: Mental Health and Well-Being Among University Students). The results of the present online pilot study are also in accordance with the findings of very recent studies. Similar to the present study, these studies examined the mental health and well-being of university students using standardized self-report measures rather than open-ended questions (e.g., surveys). Further, similar to the present study, these studies have been conducted among university students without any history of mental disorders (e.g., Farrer et al., 2016). This is also true of the all-female sample of the laboratory pilot study because they had no history of health complaints or mental disorders. Overall, this sample of female university students reported lower levels of depression than the online sample (i.e., the scores that 16.7% of the 30 female students obtained on the BDI-II were indicative of low to moderate depressive symptoms), and 30% of them reported high levels of state anxiety (i.e., when compared to 41.83% of the participants of the online study sample). These findings (i.e., lower levels of depression and state anxiety) may be valid only among undergraduate students and freshmen because 26 of the 30 female participants were first-year undergraduate students.

With regard to perceived stress, all the students reported experiencing chronic stress due to uncertainty (i.e., financial uncertainty, job uncertainty, uncertainty about other domains of life such as career, family, and friends) and excessive demands (e.g., related to work and social life). These two types of stressors might be major contributors to the consistently high levels of stress that university students experience throughout the entire duration of their academic program. Accordingly, all the participants of the online study reported experiencing several psychosomatic symptoms. With regard to their quality of life, they were more satisfied with their environments and physical health than with their well-being and social relationships. This finding supports the contention that university students are sensitive to and aware of the psychological factors that promote and impair their well-being and mental health.

Further research is needed to determine the generalizability of the present findings to the larger population of university students (e.g., those in other countries). Nevertheless, the present findings are true of those without any history of health problems. Thus, the findings of the present series of pilot studies may also be true of university students without a history of psychiatric, mental, and somatic disorders.

### Physical Activity, Mental Health, and Well-Being Among University Students

Among the male and female participants of the online study, none of whom had any history of psychiatric disorders, self-reported engagement in regular physical activity at T1 was significantly and negatively correlated with self-reported depression, trait and state anxiety, psychosomatic symptoms, and body dissatisfaction. Furthermore, physical activity was positively correlated with positive affect and the following quality of life domains: satisfaction with physical health, well-being, and social relationships.

The present findings support the contention that there is a relationship between regular physical activity, mental health, and well-being among university students. Importantly, this relationship was already confirmed using T1 measurements. Therefore, this relationship is independent of the effects of the exercise intervention and any of the control interventions that were used in this study. This suggests that regular physical activity significantly enhances mental health and well-being among university students whose average time spent sitting is approximately 7.5 h per day.

These correlational results cannot be treated as evidence of causal relationships, but the aforementioned correlations suggest that regular physical activity protects university students from mental health problems. Indeed, it was associated with lower levels of depression, anxiety, and psychosomatic stress and better quality of life across the following domains: physical health, well-being, and social relationships. This interpretation concurs with past findings, which suggest that physically active people report lower levels of depression and anxiety and that physical activity, in general, can prevent or alleviate depression, anxiety, and stress symptoms (e.g., Taylor et al., 1985; Paluska and Schwenk, 2000; Penedo and Dahn, 2005). Moreover, short-term exercise interventions may have beneficial effects on the mental health and well-being of university students. The results of the pilot studies, which are discussed in the following sections, partially support this interpretation.

### Effects of the Short-Term Aerobic Exercise Intervention on Mental Health and Well-Being

The 6 week low- to moderate-intensity aerobic exercise intervention significantly decreased depressive symptoms among the female and male participants of the online study sample (i.e., between T1 and T2). Among those assigned to the aerobic exercise intervention condition, 73.68% demonstrated improvements in depressive symptoms between T1 and T2. The aerobic exercise group scored at T1 on average two points higher on the BDI-II than the expressive writing group or the waiting list. However, this difference was not statistically significant. From a clinical perspective, the mean scores obtained by the aerobic exercise group participants at T1 were indicative of minimal depressive symptoms. In contrast, the scores obtained by the participants of the other two groups were indicative of a relative absence of depressive symptoms at T1. Therefore, short-term aerobic exercise interventions may be particularly effective in reducing the severity of depressive symptoms among university students who already report depressive symptoms for a constant time at T1. The validity of this speculation should be tested in future studies using larger cohorts of university students.

In addition, the 6 week low- to moderate-intensity aerobic exercise intervention resulted in significant improvements in perceived stress between T1 and T2 (within-subjects comparisons). Notably, perceived stress due to uncertainty increased between T1 and T2 (i.e., effect of time), but this change was less pronounced among the participants assigned to the aerobic exercise intervention group. This supports the contention that engagement in aerobic exercise serves as a buffer against psychological stress among university students (e.g., Kim and McKenzie, 2014; Magalhaes, 2016). This effect was specific to the aerobic exercise intervention. Thus, among university students, aerobic exercise appears to be more efficient in regulating perceived stress than other interventions such as cognitive interventions (e.g., expressive writing). However, the 6 week aerobic exercise intervention did not alleviate the psychosomatic symptoms of perceived stress. Further, those assigned to the exercise intervention group did not demonstrate significant changes in quality of life when compared to the waiting list control and expressive writing intervention groups. The participants assigned to the exercise and expressive writing intervention groups reported that the intervention that they had received (i.e., exercise or expressive writing) alleviated their stress and enhanced their well-being. However, the perceived contributions of their respective interventions to stress regulation and well-being were greater among those assigned to the exercise intervention group than among those assigned to the expressive writing intervention group. Moreover, as mentioned earlier, only the 6 week exercise intervention was effective in decreasing the overall level of perceived stress, especially stress caused by uncertainty.

There were no significant changes in anxiety scores between T1 and T2 (i.e., within-subjects comparisons) in any of the three groups. However, state anxiety scores at T2 were lower among those assigned to the exercise intervention group than among those assigned to the expressive writing intervention or waiting list control group. Recent meta-analytic studies have found that even a single bout of exercise can significantly reduce state anxiety (Ensari et al., 2015). However, the anxiolytic effects of aerobic exercise become evident only after a minimum of 10 weeks of regular engagement in 21 min aerobic exercise sessions (e.g., Petruzzello et al., 1991).

Past findings on the dose-effect relationship between aerobic exercise and mental health parameters are less consistent. Health-related exercise recommendations (e.g., WHO, ACSM) are disproportionately founded upon their physical health benefits than on their contribution to mental health and well-being. With regard to depression, it is well established that regular exercise (aerobic and anaerobic exercise) has marked antidepressant effects on patients with clinical manifestations of depressive symptoms (for an overview, see Morres et al., 2019). However, little is known about the antidepressant effects of exercise on mild, minimal, and moderate subclinical depressive symptoms. Although a small sample was used in the laboratory study, a comparison of the results of the present series of pilot studies revealed that six but not 2 weeks of regular engagement in low- to moderate-intensity aerobic exercise can significantly alleviate the subclinical depressive symptoms of healthy university students (both men and women) without a history of depression. These effects were evident only after regular engagement in low- to moderate-intensity aerobic exercise for 6 weeks. This observation concurs with recent recommendations, which underscore the need to not only explore the therapeutic effects of low- to moderate-intensity aerobic exercise among clinical populations but also test the effectiveness of aerobic exercise in promoting mental health and well-being. The present series of pilot studies on mental health, well-being, and the effects of physical activity and short-term low- to moderate-intensity aerobic exercise on university students are first steps in this direction.

One of the limitations of the present pilot studies is the use of small samples. However, a pre-post design and a randomized controlled trial and well-controlled online and laboratory study protocols were used. Therefore, this study can serve as a model for future studies. This design allowed to disentangle the quasi-experimental effects of self-reported physical activity from the effects of the short-term aerobic exercise intervention on mental health and well-being. At the same time, it allowed to compare the effects of the short-term aerobic exercise to those of stress-relieving cognitive (i.e., expressive writing) and other types of exercise (i.e., motor coordination) interventions. Expressive writing has been used in cognitive behavioral therapy, in cognitive stress-prevention and self-regulation programs in students and patient samples including multiple or single sessions (e.g., Baikie and Wilhelm, 2005; Lepore, 2006; Herbert et al., 2019). The exercises included in the motor coordination intervention (i.e., balance, agility, and coordination) were selected in accordance with the recommendations of the ACSM (Garber et al., 2011; American College of Sports Medicine, 2013), which aims to promote the maintenance of motor skills and executive functions across the developmental life span and especially among older adults. The maximal duration and intensity of the motor coordination exercises were lower than those that the ACSM has recommended for motor skills-related exercise training (20–30 min per day). Nevertheless, the participants assigned to the motor coordination intervention group demonstrated significant improvements in motor balance (which was assessed using the balance-board subtest). Thus, aerobic exercise appears to be effective in enhancing the well-being of university students, and other types of exercises (e.g., motor coordination exercises) appear to have unique effects on cognitive and motor performance. In this experimental study, an all-female sample was used to examine the effects of the motor coordination and aerobic exercise interventions. Therefore, future studies should test the present findings using larger samples of university students that include both women and men. Further, future studies should use less conservative inclusion and exclusion criteria. Indeed, in the present series of pilot studies, only participants who completed the interventions were included in the sample.

#### Cardiovascular Effects

The laboratory study examined exercise-induced changes in cardiovascular fitness. At T1 and T2, resting HRV was measured using non-invasive recordings of the ECG (III-lead-ECG), which served as psychophysiological indicator of cardiovascular fitness. At rest, HRV is under sympathetic and parasympathetic (vagal) control. Because high-frequency (HF) modulations (0.15–0.4 Hz) of the R-R interval of the human heart are regulated through top-down innervations by the parasympathetic (vagal) nerve, the HRV-HF (n.u.) band of HRV is frequently used as a marker of cardiovascular fitness (e.g., Perini and Veicsteinas, 2003; Oliveira et al., 2018; Singh et al., 2018). Moreover, changes in HRV-HF (n.u.) are indicative of exercise-induced adaption in cardiovascular regulation. This would be accompanied by an increase in resting HRV-HF (n.u.) (pre-post), which in turn would suggest that regular aerobic exercise facilitates cardiac vagal tone on the heart at rest. In healthy individuals, cardiovascular fitness improves after 6 weeks of regular engagement in moderate-intensity aerobic exercise (Hottenrott, 2006). This explains why the participants of the laboratory study demonstrated no significant changes in cardiovascular fitness after the 2 week low- to moderate-intensity aerobic exercise intervention. Interestingly, in the all-female sample, resting HRV-HF (n.u.) was negatively correlated with self-reported state anxiety and negative affect and marginally significantly and negatively correlated with self-reported depressive symptoms at T1. Moreover, at T2, the cardiovascular fitness of the participants assigned to the two exercise intervention groups was better than those assigned to the waiting list control group. This finding underscores the role that cardiovascular fitness plays in the mental health and well-being of female university students. Replication studies should aim to further examine the validity of these findings.

## Conclusion and Future Outlook

The present series of pilot studies answer four major questions. None of these questions has been addressed in sufficient detail in previous studies. The present findings regarding the mental health and well-being of university students, which were derived using standardized questionnaires, are alarming because they concur with the results of larger online surveys. In this study, physical activity was significantly related to self-reported depressive symptoms, anxiety, positive affect, and quality of life, irrespective of the gender of the participants and the academic degrees that they were pursuing. These findings underscore the important role that physical activity plays in the well-being of university students. Furthermore, they confirm that regular engagement in low- to moderate-intensity aerobic exercises for 6 weeks is effective in alleviating subclinical depressive symptoms and perceived stress among university students without a history of depression and health concerns. Short-term aerobic exercise interventions may be more effective in promoting the mental health and well-being of students than cognitive interventions are. Therefore, low- to moderate-intensity exercise (e.g., as the one used in the present study) should be incorporated into daily university schedules. Delivering the exercise programs used in this study as online video tutorials may allow university students to practice these exercises in the comfort of their own homes and at a time that is convenient to them. This is also a cost-effective means of reaching less intrinsically motivated students and increasing their likelihood of engagement in regular exercise. With regard to the role of monetary incentives in motivating individuals to participate in studies on mental health and well-being, women have been found to be more likely to participate in online studies, especially those that are related to health. Moreover, high monetary incentives and incentives with low probabilities of payoffs (e.g., lotteries or raffles) have positive effects on study compliance (e.g., Zutlevics, 2016; Vlaev et al., 2019). Different incentives were offered in the two pilot studies (i.e., online study: raffle vs. laboratory study: individual monetary rewards), but they may have had similar effects on potential participants’ motivation to participate. In this regard, offering evidence-based exercise programs in classrooms (e.g., by incorporating exercise breaks into weekly lectures and courses and implementing them either directly or through online modules) may be effective in increasing university students’ motivation to increase their regular physical activity behaviors, despite their overscheduled work days. Despite constant progress within the field of health management and a wealth of available exercise options, corresponding initiatives to provide structured health management exercise interventions to university students remain sparse (e.g., Brown, 2018). The present findings underscore the need to implement exercise interventions in universities to promote mental health and well-being among university students in the short (i.e., during the course of their academic program) and long term.

## Data Availability Statement

The data supporting the conclusions of this article will be made available by the corresponding author, without undue reservation, to any qualified researcher.

## Ethics Statement

The experimental protocol, design of the exercise intervention, and its laboratory assessment were reviewed and approved by the local ethics committee of Ulm University^4^. The participants of the online study and the laboratory study provided their written informed consent to participate in the study. Written informed consent was obtained from the individual for the publication of any potentially identifiable images or data included in this article.

## Author Contributions

CH drafted and wrote the manuscript and revised it for intellectual content. CH developed, conceptualized and designed the two studies, the experimental designs, the exercise training programs and the RCT for the exercise and cognitive intervention reported in this manuscript (online and laboratory design). CH developed the standardized protocol for HRV measurement at rest. CH supervised and contributed to data collection and data preprocessing (online and laboratory study). CH performed data methods and data interpretation. CH, FM, and VG performed the statistical data analysis of the online study. FM performed the piloting of the *n* = 10 sample for estimation of max. heart rate. CH and CW performed the statistical data analysis of the laboratory study. Tables and Figures were created by FM, CW and revised by CH. English translations for the exercises in the Tables 1A,B, 5 were provided by FM and CW.

## Conflict of Interest

The authors declare that the research was conducted in the absence of any commercial or financial relationships that could be construed as a potential conflict of interest.

## Footnotes

1 Additionally, 5 participants were interested in participating in the online study, but they did not complete any of the study measures.

2 https://glneurotech.com/bioradio/

3 The 12 min aerobic (endurance) exercise subtest of the Beuker-Stemper test battery was not used in the laboratory study. Instead, a motor balance test was used (i.e., participants were required to stand on a dynamic board and maintain their balance for as long as possible).

4 https://www.uni-ulm.de/einrichtungen/ethikkommission-der-universitaet-ulm/
